# Navigating fluoride hesitancy: mapping the evidence base for fluoride-free toothpaste alternatives

**DOI:** 10.1007/s00431-026-07176-y

**Published:** 2026-06-18

**Authors:** Alexander J. Wong, Christopher C. Donnell

**Affiliations:** 1 Department of Paediatric Dentistry, Charles Clifford Dental Hospital, Sheffield Teaching Hospitals NHS Foundation Trust, 76 Wellesley Road, Sheffield, S10 2SZ UK; 2 Academic Unit of Oral Health, Dentistry and Society, School of Clinical Dentistry, University of Sheffield, Sheffield, UK

**Keywords:** Fluoride hesitancy, Fluoride-free toothpaste, Caries prevention, Paediatric dentistry, Remineralisation, Hydroxyapatite

## Abstract

This study aims to synthesise and interpret evidence relating to fluoride-free toothpaste alternatives for caries prevention, examining how these products have been studied, the outcomes used to evaluate them, and how this evidence may inform discussions with children and families. A scoping review was undertaken. Electronic searches of major bibliographic databases identified peer-reviewed studies published between January 2020 and March 2026 evaluating fluoride-free toothpaste formulations. Data were extracted on intervention type, study design, outcome measures, and population characteristics and synthesised narratively with emphasis on patterns of evidence generation rather than comparative effectiveness. A total of 122 studies were included, comprising predominantly laboratory and mechanistic investigations, with only 20 studies providing direct paediatric evidence. Fluoride-free formulations were associated with antimicrobial activity, biofilm modulation, and surrogate remineralisation outcomes. Direct assessment of clinical caries outcomes was uncommon, and substantial heterogeneity was observed in formulation composition, study design, and outcome selection.

*Conclusion*: Fluoride-free toothpaste formulations demonstrate measurable biological effects, but evidence is dominated by surrogate outcomes and short-term study designs. While hydroxyapatite-based products have the most consistent evidence base, no fluoride-free category currently has evidence comparable to fluoride for long-term caries prevention in children.

**Table:** 

**What is Known:** • *Fluoride toothpaste has strong evidence for long-term caries prevention.* • *Fluoride-free alternatives are increasingly used, but existing evidence is largely based on surrogate or short-term outcomes.*
**What is New:** • *Across formulations, observed effects are driven more by study design, delivery context, and outcome selection than by specific ingredients.* • *No fluoride-free category shows equivalent clinical evidence to fluoride for long-term caries prevention, although hydroxyapatite demonstrates relatively more consistent, yet still limited data.*

**Supplementary Information:**

The online version contains supplementary material available at 10.1007/s00431-026-07176-y.

## Introduction

Fluoride remains one of the most effective and extensively studied agents for the prevention of dental caries. High-quality systematic reviews and meta-analyses have consistently reported substantial and clinically meaningful reductions in caries incidence associated with fluoride use, including a clear dose–response relationship for fluoride toothpastes in paediatric and adolescent populations [1]. Overviews of Cochrane systematic reviews further conclude that topical fluoride therapies are associated with clear decreases in caries incidence in both primary and permanent dentitions [2]. Longitudinal evaluations and natural experiments show that reductions or withdrawal of fluoride exposure are followed by increases in caries experience, particularly among children, underscoring its central role in population-level prevention strategies [3–6].

Despite this evidence, clinicians increasingly encounter caregivers who express reluctance or refusal regarding fluoride use for their children. Qualitative research indicates that fluoride hesitancy is rarely driven by doubts about effectiveness but instead reflects concerns related to perceived toxicity, long-term exposure, autonomy, and trust in health institutions [7, 8]. It also reflects broader patterns of parental decision-making in child health, where preventive recommendations may be accepted, modified, or declined. More recent mixed-methods and cross-sectional studies suggest that clinicians frequently report uncertainty and variability in counselling approaches when engaging with fluoride-hesitant families, highlighting practical challenges in prevention-focused communication [9–11].

In response to these concerns, many families adopt fluoride-free toothpaste alternatives rather than disengaging from oral health prevention. Market analyses and qualitative studies describe increasing uptake of products marketed as “natural”, “organic”, “vegan”, or “biological”, often accompanied by claims related to antimicrobial activity, enamel strengthening, or remineralisation [12–15]. In parallel, dental research has explored a wide range of fluoride-free compounds, including mineral-based, herbal, and synthetic bioactive agents proposed to modify oral biofilms or support enamel integrity [16]. Laboratory and early-phase clinical studies report diverse mechanistic effects, but reviews consistently highlight substantial heterogeneity in study design, outcome selection, and delivery context, complicating interpretation and translation to routine care [17–19].

Against this backdrop, there is a need for synthesis that does not seek to adjudicate the fluoride debate, but to clarify how evidence on fluoride-free toothpaste alternatives has been generated and how it may be interpreted in practice. This review provides a critical synthesis of fluoride-free toothpaste alternatives that families may wish to discuss or may already have adopted, highlighting recurring themes within a heterogeneous evidence base. By focusing on active ingredients used within toothpaste formulations and interpreting findings through a paediatric clinical lens, it aims to support proportionate, evidence-informed clinician-family discussions grounded in what is known and what remains uncertain.

Accordingly, this review aimed to synthesise and interpret the evidence relating to active ingredients used in fluoride-free toothpaste alternatives for caries prevention, examining how these agents have been studied across formulation, delivery context, and outcome measures, and considering how this evidence may be interpreted within routine paediatric dental practice.

## Methods

### Review design and methodological framework

This study was conducted as a scoping review to examine the breadth, nature, and characteristics of evidence relating to fluoride-free caries-preventive interventions. A scoping review approach was selected to accommodate the expected heterogeneity of interventions, study designs, and outcome measures [20]. The review question was structured using the Population, Concept and Context (PCC) framework. The population was defined broadly as evidence involving children, adults, mixed populations, laboratory models, or in situ models relevant to fluoride-free toothpaste use. The concept was fluoride-free toothpaste alternatives and active ingredients proposed for caries prevention or enamel protection. The context was routine oral health prevention, with particular attention to how evidence may inform paediatric dental practice and clinician-family discussions.

Rather than estimating comparative effectiveness or undertaking quantitative synthesis, the review aimed to map how evidence for fluoride-free alternatives has been generated and the contexts in which these interventions have been investigated. Scoping review methodology informed the identification and organisation of evidence, recognising the diversity of formulations, proposed mechanisms of action, and outcome measures characterising this field [21, 22].

The review was informed by established methodological frameworks and reported in accordance with PRISMA-ScR guidance [23]. Narrative synthesis was used to interpret patterns across the included literature, with a focus on how fluoride-free alternatives are investigated and the types of outcomes reported.

### Information sources and search strategy

The search strategy was developed in collaboration with an academic health librarian to support structured identification of relevant literature. Electronic searches were conducted across MEDLINE (via Ovid), PubMed, Embase, Scopus, Web of Science, and the Cochrane Library. Searches were restricted to studies published between January 2020 and March 2026 to reflect contemporary formulations, current clinical practice, evolving patterns of fluoride hesitancy, and demand for alternatives.

The strategy was intentionally broad, incorporating a range of product types, active ingredients, and terminology associated with fluoride-free and alternative oral care approaches. Search terms combined concepts relating to fluoride alternatives, caries prevention, and oral health, using controlled vocabulary and free-text terms adapted for each database.

Reference lists of included studies and relevant reviews were screened to identify additional eligible publications. Searches were limited to peer-reviewed studies, with no restrictions on study design or population at the search stage. The Ovid MEDLINE strategy is detailed in Box 1 and was adapted for use across the remaining databases.

**Table:** **Box 1** Ovid MEDLINE search strategy

1 (fluoride-free or fluoride-alternative* or fluoride-substitute* or zero-fluoride or no-fluoride or non-fluoride).mp. 2 (fluoride adj3 hesitan*).mp. 3 (alternative* adj3 fluoride).mp. 4 (fluoride-replacement* or replacing-fluoride).mp. 5 (substitut* adj for* adj fluoride).mp. 6 (replacement adj for* adj fluoride).mp. 7 "natural oral care".mp. 8 ((natural or organic) adj2 (extracts or extract or agents or product*)).mp. 9 "essential oil*".mp. 10 ((herbal or plant) adj (extracts or agents)).mp. 11 (nano-hydroxyapatite or Nano-HAP or nanohydroxyapatite).mp. 12 (thymol or sorbitol or xylitol or chlorhexidine or eugenol or "clove oil" or arginin* or "aloe vera" or spearmint or peppermint or theobromine or neem or margosa or nimtree or "indian lilac" or "Azadirachta indica" or "tea tree oil" or "hydrated silica" or "green tea extract" or "sodium bicarbonate" or "zinc citrate" or "calcium citrate" or "diatomaceous earth" or "coconut oil" or charcoal or eucalyptus or "bamboo salt" or "licorice root extract" or "liquorice root extract" or "calcium phosphate*" or "silver nanoparticles" or "manuka honey" or stevia or organics).mp. 13 "salivary proteins".mp. 14 7 or 8 or 9 or 10 or 11 or 12 or 13 15 fluoride*.tw. 16 14 not 15 17 1 or 2 or 3 or 4 or 5 or 6 or 16 18 Mouthwashes/ 19 exp Dentifrices/ 20 exp Chewing Gum/ 21 ((lozenge* or spray* or powder* or varnish) and ("oral health" or "oral hygiene" or "dental hygiene")).mp. 22 (mouthwash* or mouth rinse* or "oral rinse" or "mouth gel" or "mouth gels" or "dental gel" or "dental gels" or "oral gel" or "oral gels" or "oral varnish" or "mouth varnish" or "dental varnish" or "tooth varnish").mp. 23 (dentifrice* or toothpaste* or "toothpowder*" or tooth-powder*).mp. 24 ("oral hygiene" or "dental hygiene" or "oral clean*" or "mouth clean*" or "tooth clean*").mp. 25 18 or 19 or 20 or 21 or 22 or 23 or 24 26 17 and 25 27 ("dental caries" or caries or carious or "tooth decay" or "dental decay" or "tooth disease*" or "oral disease*" or "dental disease*" or "oral hygiene" or "dental hygiene" or "mouth hygiene" or "preventive dentistry" or "dental prevention").mp. 28 26 and 27 29 exp animals/not humans/ 30 28 not 29 31 (dog or dogs or cat or cats or minipig* or mini-pig* or monkey* or macaque* or rat or rats or mouse or mice or rabbit* or horse* or chicken* or rooster* or "animal stud*").tw. 32 30 not 31 33 limit 32 to english language 34 limit 33 to yr="2020 -Current" 35 remove duplicates from 34	

### Eligibility criteria

Eligibility criteria were defined a priori to identify studies evaluating fluoride-free alternatives relevant to caries prevention, while allowing inclusion of heterogeneous evidence types consistent with a scoping review approach. Full inclusion and exclusion criteria are summarised in Box 2.

**Table:** **Box 2** Eligibility criteria

**Inclusion criteria** Studies were eligible for inclusion if they met the following criteria: • Evaluated fluoride-free oral interventions or biologically active agents intended to prevent dental caries or support enamel protection or remineralisation, including those studied across different delivery contexts • Examined active ingredients relevant to fluoride-free toothpaste formulations—while the review is anchored to toothpaste as the primary delivery context, studies evaluating these ingredients in alternative experimental or delivery settings (e.g. laboratory, in situ, or non-toothpaste formulations) were included where they informed understanding of their proposed mechanisms or effects • Reported caries-related outcomes or validated surrogate measures (e.g. enamel hardness, mineral loss or gain, lesion characteristics, biofilm or microbiological outcomes) • Employed laboratory, in situ, or clinical study designs, including randomised and non-randomised studies • Included paediatric, adult, or mixed populations—given the absence of age-stratified formulations in many fluoride-free toothpaste products, studies were not restricted to paediatric populations, instead the review focused on active ingredients present in toothpaste formulations used by families, irrespective of the population in which these agents were studied • Were peer-reviewed and published between January 2020 and March 2026	
**Exclusion criteria** Studies were excluded if they met any of the following criteria: • Evaluated fluoride-containing interventions without a clearly defined fluoride-free comparator • Focused exclusively on non-toothpaste delivery vehicles (e.g. mouthrinses, gels, varnishes, or other adjunctive products) evaluating active ingredients not used within toothpaste formulations • Addressed behavioural, educational, or policy-based interventions without assessment of a physical or chemical fluoride-free alternative • Did not report outcomes relevant to caries prevention or enamel integrity • Were conference abstracts, opinion pieces, editorials, or non-peer-reviewed sources • Were published outside the defined date range	

### Study selection and screening

Search results were combined into a single dataset and managed using Microsoft Excel (Mac Version 16.101.3), with duplicate records removed prior to screening. Eligibility assessment was conducted in two stages: title and abstract screening followed by full-text review of potentially eligible studies. Screening was undertaken collaboratively by the authors and applied iteratively to ensure consistent interpretation of eligibility criteria, with a subset of studies reviewed independently to support consistency. Any ambiguity was resolved through discussion until agreement was reached.

### Data extraction and synthesis

Data were extracted using a purpose-designed framework capturing intervention type, formulation characteristics, study design, outcome measures, and population features. Evidence sources were categorised by study type, including laboratory studies, in situ studies, clinical trials, observational studies, systematic reviews, narrative reviews, and other evidence syntheses. Population characteristics were recorded to facilitate interpretation of the evidence base within a paediatric context, including whether studies provided direct paediatric evidence, mixed-population evidence, adult evidence, or mechanistic evidence. The framework was piloted and refined iteratively to ensure relevance across the included literature, with the final set of variables presented in Box 3.

**Table:** **Box 3** Data extraction fields

1. Author(s) 2. Year of publication 3. Title of publication 4. Source of publication (e.g. journal) 5. Country of author(s) 6. Aim(s) or stated purpose of the study 7. Study design (e.g. laboratory, in situ, or clinical; randomised or non-randomised where applicable) 8. Study population and setting, including sample size, age group where reported, and relevance to paediatric practice (paediatric, mixed-population, adult, or mechanistic evidence) 9. Intervention and comparator characteristics, including product type and formulation where reported 10. Outcome measures assessed, including clinical outcomes and validated surrogate measures 11. Key findings relevant to caries prevention or enamel protection 12. Additional notes relevant to interpretation (e.g. contextual or methodological features)	

Extracted data were synthesised narratively to identify patterns in intervention type, formulation, study design, setting, and outcome measures. Synthesis focused on the nature and strength of available evidence rather than estimation of effect size, emphasising consistencies, areas of divergence, and evidence gaps relevant to paediatric dental practice. Secondary evidence syntheses were used to contextualise findings and were not combined quantitatively with primary studies. No formal quantitative analysis or statistical pooling was undertaken, reflecting the heterogeneity of the included evidence.

## Results

### Overview of included studies

**Fig. 1:**
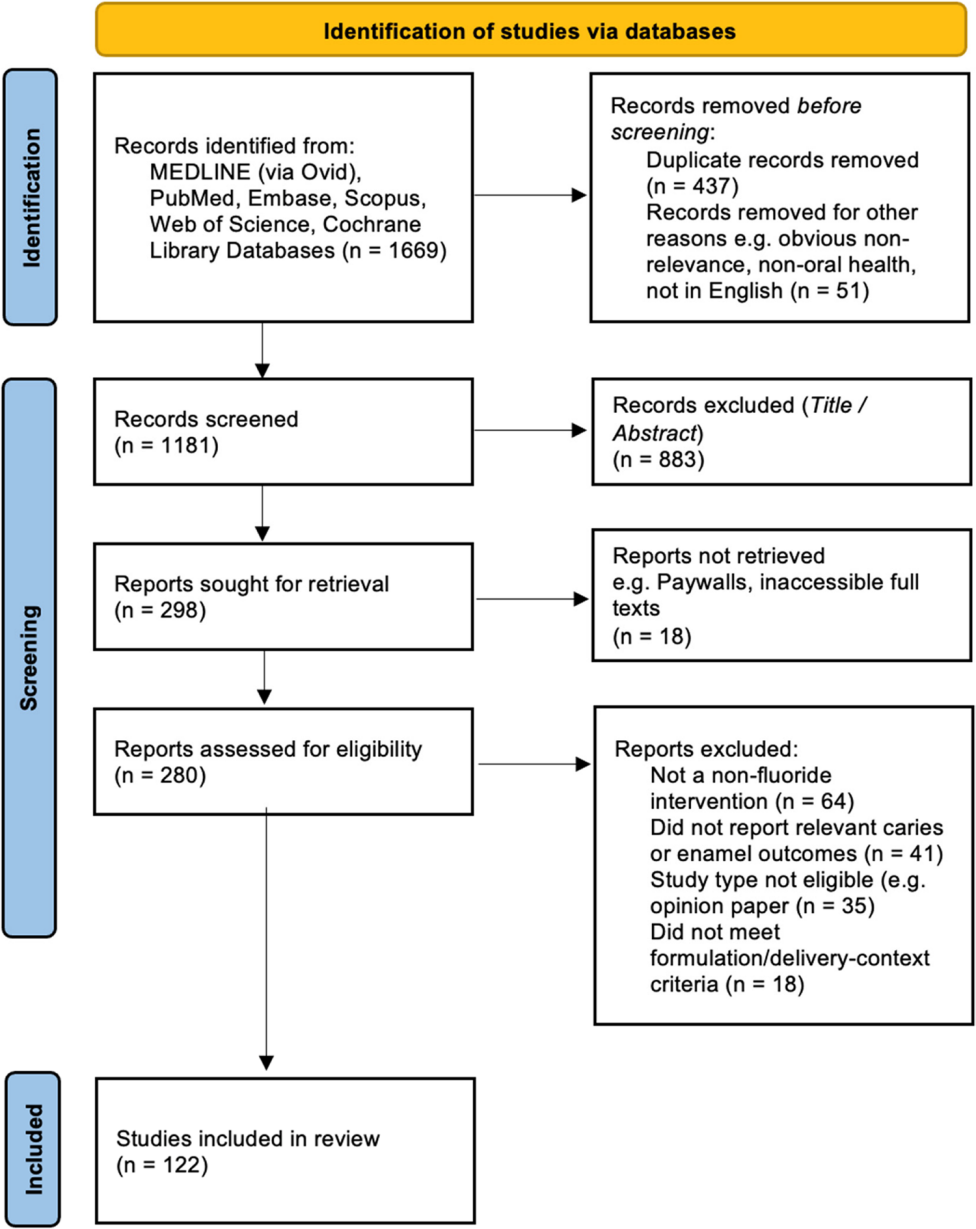
PRISMA flow diagram—study selection process

Database searches identified 1669 records, of which 122 met the eligibility criteria and were included following title and abstract screening and full-text assessment (Fig. 1). The included studies comprised 39 laboratory studies (32.0%), 32 clinical studies and trials (26.2%), 22 narrative reviews and other non-systematic evidence syntheses (18.0%), 14 systematic reviews and meta-analyses (11.5%), eight observational studies (6.6%), five in situ studies (4.1%), and two scoping reviews (1.6%). Study populations were similarly heterogeneous, comprising 20 studies providing direct paediatric evidence (16.4%), 15 involving adult populations (12.3%), 29 drawing on mixed populations (23.8%), and 58 representing mechanistic evidence (47.5%).

Interventions varied widely, encompassing mineral-based, herbal, bioactive glass, and other synthetic or naturally derived agents evaluated in commercial and experimental formulations, with comparators including fluoride-containing toothpastes, placebo, or no-treatment controls [24–41].

Table 1 summarises study design, outcome domains, paediatric relevance, and key interpretive constraints across intervention categories, with detailed study characteristics provided in Supplementary File S1.

**Table 1:** Summary of evidence characteristics, outcome domains, and interpretive constraints across fluoride-free toothpaste categories

Intervention category	Predominant study designs	Typical outcome domains assessed	Paediatric relevance	Key interpretive constraints	References
Mineral-based (e.g. hydroxyapatite)	Laboratory; in situ; short-term clinical	Remineralisation proxies; surface hardness; lesion depth	Direct (limited); extrapolated	Short follow-up; surrogate outcomes; formulation variability	[24, 25, 29, 30, 33, 34, 36, 41–61]
Herbal/botanical formulations	Laboratory; in situ	Antimicrobial activity; biofilm modulation	Indirect	In vitro dominance; non-standardised extracts; outcome heterogeneity	[27, 38, 58, 62–94]
Bioactive glass/synthetic agents	Laboratory; in situ	Mineral deposition; enamel interaction; pH buffering	Largely absent	Experimental delivery contexts; mechanistic focus	[40, 79, 95–100]
Sugar alcohol-containing toothpastes	Short-term clinical; laboratory	Plaque indices; bacterial counts; surrogate caries markers	Mixed	Behavioural confounding; limited caries endpoints	[35, 37, 96, 101–106]
Other emerging formulations	Predominantly laboratory	Mechanistic or antimicrobial endpoints	Absent	Early-phase evidence; translational uncertainty	[9, 16, 26, 28, 31, 32, 39, 56, 61, 107–146]

### Types of fluoride-free alternatives identified

A wide range of fluoride-free alternatives were identified and grouped according to primary active component or mechanism of action across multiple study designs. Across categories, many formulations contained multiple active components, limiting attribution of observed effects to individual ingredients.

#### Hydroxyapatite-based formulations

Hydroxyapatite-based formulations were among the most frequently investigated alternatives, studied across laboratory, in situ, and clinical contexts [24, 25, 29, 30, 42]. Microcrystalline and nano-hydroxyapatite variants, including modified formulations, were primarily evaluated using remineralisation-related outcomes such as surface microhardness, lesion characteristics, and enamel mineral dynamics [25, 36, 40, 43–47].

Clinical investigations included randomised trials in paediatric and adult populations with follow-up ranging from several months to 2 years [33, 34, 36, 48–50]. Mechanistic and translational studies provided complementary evidence on enamel interactions and biofilm-related outcomes [25, 29, 30, 40, 42, 43, 107]. In addition to primary studies, several evidence syntheses and reviews focused specifically on hydroxyapatite-based, fluoride-free toothpastes, reflecting sustained research attention to this intervention class [41, 51–53, 147]. Across study designs, these formulations were associated with reduced enamel demineralisation and improved surface microhardness and in some cases reported non-inferiority to fluoride comparators for early caries outcomes.

#### Bioactive glass-containing products

Bioactive glass formulations represented a smaller category, most commonly incorporated into toothpaste matrices. Evidence focused on ion release mechanisms and associated remineralisation under laboratory and in situ conditions [25, 40].

Clinical evidence included a randomised trial in a paediatric population, supported by laboratory studies demonstrating enamel mineralisation and surface changes using surrogate measures [40, 95]. Across study designs, these formulations were associated with remineralisation-related outcomes, although evidence remained limited and predominantly mechanistic.

#### Arginine-containing toothpastes

Arginine-containing formulations were investigated as fluoride-free or adjunctive approaches targeting plaque biofilm ecology [37, 96, 101, 102]. Evidence included paediatric clinical trials assessing caries incidence and microbiological outcomes, alongside laboratory studies examining biofilm composition [37]. Across studies, arginine-based formulations were associated with changes in plaque ecology and reductions in cariogenic markers, although evidence for long-term clinical outcomes remains limited.

#### Xylitol-based interventions

Xylitol-based toothpaste interventions were represented by a limited number of clinical studies and secondary analyses, largely in paediatric populations. Outcomes focused primarily on microbiological markers, with fewer studies reporting clinical caries measures [35, 103].

Secondary syntheses contextualised these findings within the broader xylitol literature [104–106]. Overall, evidence for xylitol-containing toothpastes remains limited in volume and scope, with minimal investigation of enamel-level or mechanistic outcomes.

#### Herbal, mineral, and naturally derived compounds

A heterogeneous group of formulations incorporated herbal extracts, plant-derived compounds, and alternative mineral agents [38, 62, 63]. These were evaluated across laboratory and short-term clinical studies, with outcomes primarily related to antimicrobial activity, biofilm modulation, and plaque-associated measures [64, 65, 107–110].

Additional agents included theobromine, zinc salts, sodium bicarbonate, charcoal, and nanoparticle-based systems, with studies predominantly reporting outcomes such as antimicrobial effects and short-term plaque changes [97, 111–117]. A small number of studies explored biologically active compounds in alternative delivery contexts, including dietary interventions such as pomegranate [66], reflecting broader interest in naturally derived anticariogenic agents. Across this category, evidence was highly heterogeneous, with limited reporting of long-term caries outcomes and minimal translation to consistent clinical endpoints.

#### Evidence syntheses and reviews

A limited number of secondary evidence syntheses were identified, predominantly focused on hydroxyapatite-based formulations [41, 52, 53]. These integrated findings across study designs while highlighting ongoing uncertainty regarding clinical effectiveness relative to fluoride comparators [54].

Syntheses addressing other alternatives were fewer and more heterogeneous, often incorporating toothpaste-based interventions within broader oral health contexts rather than evaluating them in isolation [67, 68, 117–120].

### Study design and level of evidence

The evidence base was dominated by laboratory studies employing enamel or dentine models, pH-cycling protocols, surface microhardness testing, and biofilm assays to evaluate demineralisation, remineralisation, and antimicrobial effects under controlled conditions [25, 26, 42, 64, 102, 121, 122]. In situ studies were fewer, using intra-oral appliances to assess short-term enamel and biofilm responses following exposure to toothpaste formulations in the oral environment [31, 110].

Clinical investigations were limited and heterogeneous, comprising randomised controlled trials, cohort studies, and short-term interventions across adult, paediatric, and mixed populations, with follow-up typically restricted to weeks or months and few extending beyond one year [34, 49]. Overall, the evidence base reflects a gradient from mechanistic laboratory research to limited clinical evaluation, with higher-level evidence from adequately powered, long-duration trials remaining scarce [33, 34, 36].

### Intervention formulations and delivery vehicles

Published research reported substantial heterogeneity in formulation composition, material design, and delivery context, with a strong emphasis on experimental or prototype formulations rather than established commercial products [45, 55, 56, 69, 98].

Nanotechnology-enabled formulations were also represented, including toothpastes incorporating metal nanoparticles, plant-derived nanomaterials, or encapsulated bioactives, most commonly evaluated using laboratory-based antimicrobial or remineralisation assays [115, 116, 123, 124]. Variation in delivery context was evident, with studies employing toothpaste suspensions, modified application protocols, or enamel and biofilm models to isolate formulation effects [110, 125, 126]. Herbal and plant-derived formulations were typically evaluated as carrier matrices for bioactive compounds in laboratory or short-term clinical contexts, with limited reporting of long-term use or standardised brushing behaviour [70–73].

### Outcome measures used

Outcome assessment was highly heterogeneous, spanning microbial, physicochemical, and clinical domains. Most studies prioritised antimicrobial and antibiofilm outcomes, particularly changes in *Streptococcus mutans*, alongside alternative measures of remineralisation such as surface microhardness and lesion characteristics [35, 38, 74–78, 107, 127].

Direct clinical caries outcomes were infrequently reported. Where assessed, measures were typically limited to plaque indices, white spot lesions, or biochemical markers, rather than longitudinal caries incidence [24, 26, 31, 33, 34, 36, 40, 57, 58, 79, 80, 99, 128–131]. Survey-based and observational studies instead reported practitioner awareness, acceptability, or use of fluoride-free toothpaste products [81, 132].

### Summary of key findings across intervention types

Across intervention categories, findings clustered within a limited number of outcome domains despite substantial heterogeneity in formulation composition and study design. Reported effects were most commonly related to antimicrobial activity, biofilm modulation, and alternate measures of remineralisation, rather than long-term clinical caries outcome [24, 31, 59, 60].

Herbal and plant-derived formulations were primarily associated with antimicrobial and antibiofilm effects, while mineral-based and biomimetic agents, including hydroxyapatite, were more consistently linked to remineralisation-related outcomes [49, 59–61, 82–88].

Clinical caries incidence was typically measured over relatively short follow-up periods or through surrogate indicators [33, 49]. Nanotechnology-enabled and hybrid formulations were largely evaluated using mechanistic or proof-of-concept outcomes within laboratory or early translational contexts [115, 133–137].

### Paediatric relevance of the evidence base

Direct paediatric evidence was limited, with only 20 of the 122 included studies conducted solely with children. These studies typically employed short-term clinical or observational designs and focused on outcomes such as bacterial counts, plaque indices, or early lesion changes [33, 35, 49, 111].

Much of the evidence base inferred paediatric applicability from laboratory or mixed-population studies, particularly for herbal, mineral, and emerging bioactive formulations, where outcomes were primarily microbiological or physicochemical [89–92]. Reviews frequently referenced paediatric relevance, but underlying data were often derived from adult or in vitro research [93, 138–140].

Where children were studied directly, investigations were generally context-specific and reported surrogate or descriptive outcomes rather than intervention-driven caries prevention. Overall, paediatric relevance was more often assumed than directly evaluated within fluoride-free toothpaste research [141–146].

## Discussion

This scoping review synthesises a broad and heterogeneous body of literature examining the evidence underpinning fluoride-free toothpaste formulations, revealing consistent patterns in how these products, and the ingredients they contain, are studied and how their effects are reported. This review does not position fluoride-free alternatives as equivalent or superior to fluoride-containing toothpastes. The evidence base does not permit reliable attribution of effects to individual active ingredients, particularly in multi-component formulations, but instead clusters around recurring study designs and outcome domains, particularly antimicrobial and biofilm-related outcomes, and surrogate remineralisation measures. This pattern likely reflects methodological feasibility as much as compound-specific effects. By focusing on toothpaste formulations used by families, this review synthesises what is currently known and where important uncertainties remain about these products, particularly in relation to routine use in children.

The body of literature examining fluoride-free toothpaste formulations is best understood as emerging from fluoride hesitancy as a lived clinical and social phenomenon, rather than from scientific uncertainty regarding fluoride efficacy. A substantial programme of work led by Chi and colleagues has reported that caregivers who decline fluoride do so for multifactorial reasons, including concerns about perceived toxicity, uncertainty regarding long-term exposure, preferences for natural products, and a strong emphasis on autonomy and parental responsibility in preventive decision-making [7, 8, 10, 11, 148]. These findings are echoed across diverse populations and methodologies examining caregiver beliefs, trust in professional advice, and risk perception around fluoride use [9, 12–14, 131, 149–151]. Fluoride hesitancy does not typically manifest as disengagement from oral health practices; rather, caregivers frequently substitute fluoride toothpaste with fluoride-free alternatives perceived as safer, more natural, or more congruent with broader wellness identities [15, 152, 153]. Unlike fluoride-containing toothpastes, where formulations are commonly stratified according to age and fluoride concentration, many fluoride-free products marketed to children and adults utilise broadly similar active ingredient profiles, with differences often relating primarily to flavouring, branding, or product presentation. Consequently, clinicians are frequently required to discuss products used by children despite the supporting evidence being derived largely from adult, mixed-population, or laboratory-based research. This substitution behaviour occurs within a complex information environment shaped by media reporting, professional commentary, commercial marketing, and digital health discourse, where fluoride-related narratives are variably framed and contested [154–158]. Within this context, research attention directed towards fluoride-free toothpaste formulations reflects a pragmatic attempt to characterise the products families are already using, rather than an effort to establish equivalence with fluoride-containing toothpaste. The literature reviewed here therefore responds to patterns of parental choice and information exposure, not to equipoise in preventive effectiveness.

The focus on toothpaste formulations represents a conceptual boundary within this review, reflecting their role as routine, home-based preventive interventions embedded within everyday toothbrushing practices, including in paediatric populations. Toothpaste is uniquely integrated into daily oral hygiene behaviours, where effectiveness depends not only on formulation chemistry but also on compliance, frequency, and cumulative exposure. This aligns with qualitative literature demonstrating that parental preventive decisions are enacted within the home environment, where products must integrate into established routines rather than require additional supervision or procedural complexity [7, 8]. Much of the evidence underpinning these formulations, however, is generated outside toothpaste-specific contexts, including laboratory models, adult populations, and alternative delivery formats. While some studies approximate habitual use conditions, many evaluate bioactive agents under controlled or extended exposure scenarios that differ from routine toothbrushing [29, 32–34, 36, 159]. Anchoring the review to toothpaste formulations therefore allows findings to be interpreted within a shared and ecologically valid behavioural context, while recognising that the underlying evidence base does not consistently reflect real-world patterns of use.

The dominance of antimicrobial, biofilm-related, and remineralisation outcomes reflects the methodological structure of the evidence base rather than a hierarchy of clinical importance. Most studies employ laboratory or in situ designs that enable controlled exposure, standardised substrates, and short observation periods, making such outcomes accessible and reproducible [18, 25, 38, 43, 46, 51, 64, 68, 107, 117, 118, 121, 159]. Similar outcome domains recur across diverse intervention categories, including mineral-based, herbal, and nanoparticle-enabled formulations, often using comparable experimental platforms despite differing proposed mechanisms of action [41, 51, 52, 54, 106]. Even within clinical and in situ studies, surrogate endpoints are typically favoured over direct disease outcomes, with reported measures frequently including mineral gain, plaque indices, microbial shifts, and enamel surface changes [33, 34, 107]. These constraints reflect limitations in study duration, sample size, and ethical considerations, particularly in paediatric populations, and limit the extent to which formulation-specific effects can be isolated [33, 34, 36, 40, 49, 53, 101, 111, 119, 120, 159]. Observed similarities across intervention types are likely to reflect convergence in study design and measurement strategy rather than true equivalence in preventive effectiveness. Interpretation of this literature therefore requires careful distinction between mechanistic plausibility, short-term biological effects, and reported clinical outcomes.

In paediatric contexts, interpretation of the available literature underpinning fluoride-free toothpaste formulations is further constrained by the interaction of biological vulnerability, behavioural mediation, and parental decision-making. Enamel in primary and newly erupted permanent teeth differs structurally from mature enamel, undergoing post-eruptive maturation that influences susceptibility to demineralisation [160]. Beyond these biological factors, caries risk is shaped by caregiver-directed behaviours, including brushing practices, supervision, diet, and broader socioeconomic influences [7, 8, 10]. These factors limit the extent to which formulation-specific effects can be isolated in study designs. As a result, studies relevant to paediatric use frequently employ short follow-up periods and surrogate endpoints within ethical and practical constraints [36, 38, 121, 159]. These limitations are compounded where fluoride-free toothpaste use is most relevant, as parental beliefs influence both product selection and adherence [9, 11]. Consequently, findings must be interpreted within a framework in which biological plausibility, behavioural implementation, and caregiver mediation jointly shape preventive impact.

An additional interpretive tension concerns whether toothpaste is conceptualised as a preventive intervention or as a delivery vehicle for bioactive compounds. Across reviews and product-facing interpretations, toothpaste is often framed in the latter sense, with emphasis on transporting antimicrobial or remineralising agents under controlled conditions [17, 18, 161]. Within this framing, toothpaste functions as a matrix for compound stabilisation and application rather than as a behavioural intervention embedded within routine oral hygiene. As a result, mechanistic outcomes, such as bacterial suppression or mineral deposition, are often considered independently of the behavioural and exposure context in which toothpaste is used [162–164]. This distinction is particularly relevant for fluoride-free formulations, where proposed mechanisms frequently derive from laboratory demonstrations that may not translate to brief contact times and variable compliance characteristic of everyday toothbrushing [19, 165]. Interpreting toothpaste as both carrier and preventive agent therefore risks overstating the relevance of mechanistic findings to real-world prevention. This contextualises why many included studies, although methodologically diverse, do not directly reflect routine toothpaste use conditions.

Taken together, this review highlights a growing and methodologically diverse evidence base demonstrating that fluoride-free formulations can produce measurable biological effects across experimental contexts. However, the predominance of surrogate outcomes and short-term, laboratory or in situ designs limits extrapolation to long-term caries prevention in routine practice, particularly for children [34, 36, 107, 159, 166]. By focusing on toothpaste formulations used by families within the context of fluoride hesitancy and parental decision-making, this review provides a framework for interpreting what is currently known while making explicit where uncertainty remains. While some formulations, particularly hydroxyapatite-based products, are supported by a more consistent body of evidence, clinical data remain limited, are predominantly short-term, and do not permit definitive comparisons with fluoride. From a clinical perspective, these findings may support more nuanced discussions with fluoride-hesitant families. Evidence of remineralisation, antimicrobial activity, or biofilm modification should not be assumed to equate to established long-term caries prevention, particularly where outcomes are derived from laboratory or short-term studies. At the same time, the limitations of the evidence base need not invalidate parental concerns or preferences regarding fluoride use. Rather, clinicians may acknowledge both the emerging evidence and its uncertainties, while helping families understand the distinction between biological plausibility and demonstrated clinical effectiveness. Among the formulations reviewed, hydroxyapatite-based toothpastes currently appear to have the most consistent body of supporting evidence, whereas evidence for many herbal, naturally derived, and emerging formulations remains comparatively limited, heterogeneous, or mechanistic in nature.

This review has several limitations. The inclusion of studies evaluating active ingredients outside toothpaste-specific contexts reflects the structure of the available published evidence but limits direct applicability to real-world use of fluoride-free toothpaste formulations. In addition, the search was restricted to studies published from 2020 onwards to reflect contemporary formulations, product availability, and patterns of use; however, this may have excluded earlier foundational studies relevant to certain active ingredients. Variation in study design, populations, and outcome measures further constrained direct comparison across intervention categories. As a scoping review, formal risk of bias assessment and quantitative synthesis were not undertaken, and findings should be interpreted accordingly. The restriction to English-language publications may have excluded relevant studies, and the search strategy, while comprehensive, may not have captured all emerging or commercially driven evidence. Finally, many included studies evaluated multi-component formulations, limiting attribution of observed effects to individual ingredients.

Future research should better align outcome selection with clinically meaningful preventive endpoints, clearly articulate the behavioural assumptions underpinning toothpaste use, and adopt study designs that reflect the cumulative and contextual nature of everyday oral hygiene practices. Consideration of age-appropriate formulation strategies, including potential dose–response relationships, may also be warranted. Such advances are essential if evidence on fluoride-free toothpaste formulations is to inform clinician-family conversations without overstating certainty or implying equivalence.

## Conclusions

This review clarifies a fragmented and often polarised literature by demonstrating that reported effects of fluoride-free toothpaste formulations are influenced by study design, delivery context, and outcome selection as much as by individual ingredients. The available evidence is dominated by surrogate outcomes and short-term study designs rather than long-term caries endpoints. While hydroxyapatite-based formulations are supported by the most consistent body of evidence among fluoride-free alternatives, clinical data remain limited across all categories and do not support equivalence with fluoride for long-term caries prevention, particularly in paediatric populations.

## Supplementary Information

Below is the link to the electronic supplementary material.

ESM 1 (DOCX 118 KB)

## Data Availability

The materials and data underpinning the findings of this review can be obtained from the corresponding author upon reasonable request.
