# Supplementary material for: Navigating fluoride hesitancy: mapping the evidence base for fluoride-free toothpaste alternatives
Source: Eur J Pediatr. 2026 Jun 18;185(7):507. doi: 10.1007/s00431-026-07176-y (PMC13279590; doi:10.1007/s00431-026-07176-y)
Supplement: Supplementary file 1 — (DOCX 118 KB) [file 431_2026_7176_MOESM1_ESM.docx]

**Table S1.** **Summary of included studies evaluating fluoride-free toothpaste formulations, including study design, population characteristics, intervention type, comparator, outcome measures, and key limitations.**

| **Author(s)** | **Year** | **Country of Author(s)** | **Study Design** | **Evidence Type** | **Setting** | **Population** | **Sample Size** | **Active Ingredient(s)** | **Formulation / Delivery Context** | **Comparator** | **Outcome Measures** | **Key Findings** | **Key Limitations** |
| --- | --- | --- | --- | --- | --- | --- | --- | --- | --- | --- | --- | --- | --- |
| Gonullu I  Devrimci EE  Kemaloglu H  Peskersoy C  Turkun M | 2026 | Turkey | Experimental in vitro comparative study | Experimental study | In vitro | Extracted human third molar teeth (enamel and dentin specimens) | n=24 teeth (12 enamel, 12 dentin; subgroups n=6) | Nano-hydroxyapatite (2.5%, 5%); potassium nitrate (5%) | Experimental dentifrice gels; twice daily application (10 min) for 7 days | Baseline vs demineralised vs remineralised; 2.5% nHAp vs 5% nHAp (both with 5% KNO₃) | Surface microhardness (Vickers); EDX (Ca, P, K, Ca/P ratio); SEM morphology | Both formulations increased microhardness and mineral content; dentin fully recovered, enamel partially; no significant difference between 2.5% vs 5% nHAp | In vitro design; no clinical hypersensitivity assessment; short duration (7 days); limited depth analysis of ion penetration |
| Yin W  Zhou Z  Huang RZ  Sun G  Zhong Y  Yang Z  Li Y  Zhang Y  Zhang P  Hu D  Mateo LR  Gao G  Lim S  Ismail AI  Zimmerman J  Garcia-Godoy B  Ryan M  Zhang YP | 2026 | China, USA | Phase 3 double-blind parallel-group randomised controlled trial | Randomised clinical trial | Clinical (multi-centre, community-based) | Children aged 10-14 years with ≥2 active caries lesions | n=6,000 (3 groups ~2,000 each) | Arginine (1.5%, 8.0%); sodium fluoride (0.32% NaF control) | Toothpaste; twice-daily brushing over 24 months | 8.0% arginine vs 1.5% arginine vs 0.32% NaF dentifrice | DMFS and DMFT (incremental caries indices at 6 mo, 1 y, 2 y) | 8.0% arginine ↓ caries vs NaF (≈25-26% reduction); 1.5% arginine non-inferior to NaF; dose-dependent effect | Potential confounding (diet, hygiene, fluoride exposure); baseline imbalance; industry funding; generalisability limitations |
| Li T  Guo H  Liu C  Jiang H  Gao S  Du M | 2026 | China | Randomised double-blind multicentre parallel-group trial | Randomised clinical trial | Clinical (kindergarten/community-based) | Children aged 3-4 years with primary dentition | n=1063 (test n=580; control n=483) | Bioactive glass (7.5% w/w); fluoride (800 ppm control) | Toothpaste; supervised twice-daily brushing (school + home) over 27 months | 7.5% BAG fluoride-free vs 800 ppm fluoride toothpaste | dmft and dmfs increment; caries incidence (12 and 27 months) | BAG comparable to fluoride; no significant differences; both showed substantial caries progression | Suboptimal fluoride comparator; high attrition; potential confounding (diet, hygiene, compliance) |
| Çetin PR, Alkan E, Tağtekin D, Çetin Aİ, Oktay NŞ. | 2026 | Türkiye | Experimental in vitro comparative study (8 groups; pH-cycling model) | Experimental study | In vitro | Extracted human molar enamel specimens | n=80 (8 groups, n=10 each) | Bioactive glass; hydroxyapatite; protein-calcium glycerophosphate; ± fluoride (1450 ppm); commercial NovaMin and hydroxyapatite formulations | Toothpaste; twice-daily brushing (30 s) with pH-cycling (14 days) | Multiple experimental vs commercial toothpastes vs base paste control | Vickers microhardness; DIAGNOdent Pen; FluoreCam (lesion size/intensity) | All active formulations improved remineralization vs control; highest microhardness in fluoride-containing hydroxyapatite and NovaMin groups; fluoride enhanced effects; no significant differences between many treatment groups | In vitro model; artificial saliva; short duration; high within-group variability; limited subsurface analysis; limited clinical generalisability |
| Ramesh R, Syam S. | 2026 | India | Randomised controlled trial (parallel-group; 12-month follow-up) | Interventional (RCT) | Community/clinical (children in non-fluoridated area) | Children aged 3-6 years with early childhood caries (ECC) | n=500 (250 intervention, 250 control; high attrition) | Pomegranate (Punica granatum) extract | Dietary intervention (1.5 oz daily; swish-and-swallow; ≥60% adherence) | Standard oral hygiene instruction (no pomegranate) | DMFT; ICDAS; salivary pH; plaque index; buffering capacity | Significant improvements in caries severity, DMFT, salivary pH, and plaque index at 6 months; benefits diminished by 12 months; early protective effect not sustained long-term | Very high dropout (~45.8%); adherence variability; COVID-19 disruption; limited generalisability; behavioural confounders; effect not sustained long-term |
| Gund MP, Lehnertz L, Hannig M, Dudek J, Pütz N, Rupf S. | 2026 | Germany | In situ experimental study (crossover, 48-hour biofilm model) | Mechanistic / experimental (in situ human model) | Laboratory-clinical hybrid (in situ oral biofilm model using splints with enamel slabs) | Healthy adult volunteers (dental students/staff) | n=6 | Hydroxyapatite (5%), fluoride, chlorhexidine (0.05%), aluminium lactate, strontium acetate, potassium chloride, papain, bromelain, hyaluronic acid (varied by paste) | Toothpaste suspensions (0.5 g paste + water; rinsing protocol without brushing) | Water (negative control); base paste; 0.2% chlorhexidine (positive control) | Biofilm coverage (%); bacterial viability (live/dead fluorescence); SEM morphology; EDX analysis | All active toothpastes reduced biofilm formation vs controls; hydroxyapatite paste showed greatest reduction in bacterial viability despite higher coverage; CHX most effective overall; enzyme-based paste showed no additional antibacterial benefit | Very small sample size (n=6); short-term (48 h); no mechanical brushing (limits real-world applicability); in situ model limits generalisability; mixed active ingredients (difficult attribution); no microbiological sequencing |
| Valdivia-Tapia AC; Lippert F; Gregory RL | 2025 | USA | Randomised experimental in vitro study (MIC/MBC, biofilm assays) | Experimental study | In vitro | Bacterial model (Scardovia wiggsiae) | Not applicable (assays in quadruplicate ×3 repeats) | Hydrogen peroxide; cetylpyridinium chloride (CPC); essential oils; organic extracts; zinc chloride; stabilised chlorine dioxide | Commercial fluoride-free mouthwashes; serial dilutions (1:3, 1:6, 1:12) | TSBS (negative control); 0.12% chlorhexidine (positive control) | MIC; MBC; planktonic viability (OD595); biofilm formation (OD490) | Hydrogen peroxide and CPC strongest antimicrobial effects; essential oils moderate dilution-dependent; organic limited; zinc chloride and chlorine dioxide weakest | In vitro design; single-species model; short-term assays; dilution not reflective of clinical use; multiple actives per formulation; no clinical outcomes |
| Valdivia-Tapia AC; Hara AT; Lippert F | 2025 | USA | Experimental in vitro factorial study (2×2×4 design; pH-cycling model) | Experimental study | In vitro | Extracted human enamel specimens with artificial caries-like lesions | n=8 per group (factorial design) | Sodium fluoride (1100 ppm); stannous fluoride; cetylpyridinium chloride (0.075%); essential oils | Toothpaste slurry (1:3) twice daily (1 min) followed by mouthwash (30 s) over 5-day pH cycling | NaF vs SnF₂ toothpaste; CPC vs EO mouthwash; DIW rinse; non-wash control | Integrated mineral loss (ΔΔZ); lesion depth (ΔL) via TMR-D (conventional and single-section) | Fluoride-free mouthwashes reduced remineralization vs non-wash; no difference between NaF and SnF₂; mouthwash type significant; technique not significant for mineral loss | In vitro design; artificial lesions; no biofilm/saliva simulation; short duration; limited clinical applicability; potential variability in specimen preparation |
| Chatzidimitriou K; Theodorou K; Seremidi K; Kloukos D; Gizani S; Papaioannou W | 2025 | Greece | Systematic review and meta-analysis (PRISMA; PROSPERO-registered) | Systematic review and meta-analysis | Clinical (included RCTs/in situ trials) | Children, adolescents, and young adults (<25 years) with initial caries lesions | n=608 total (4 included RCTs; individual studies n=50-214) | Hydroxyapatite (biomimetic/nano-HAP; typically ~6.7-10%) | Toothpaste; twice or three-times daily use; follow-up 6-18 months | HAP fluoride-free vs fluoride toothpastes (e.g. NaF, AmF, SnF₂) | DMFS/DMFT; ICDAS; lesion size; fluorescence; mineral density; risk ratio/odds ratio (meta-analysis) | No significant differences in caries development/progression between HAP and fluoride; significant improvements in lesion size and fluorescence at 6 months; meta-analysis showed non-significant RR 0.98 and OR 0.90 favouring HAP | Only 4 included studies; heterogeneity in protocols and outcomes; short follow-up; unclear risk of bias in most studies; limited high-quality RCTs; variability in HAP formulations |
| Adams SE; Cawley AK; Arnold D; Hoptroff MJ; Slomka V; Matheson JR; Marriott RE; Gemmell MR; Marsh PD | 2025 | UK | Randomised double-blind parallel-group clinical trial | Clinical (microbiome/mechanistic) | In vivo (human plaque microbiome) | Adults (18-65 years; generally healthy; ≥20 teeth) | n=115 analysed (initially 131 randomised) | Zinc citrate trihydrate (2%) | Fluoride toothpaste (1450 ppm) with zinc; twice daily for 6 weeks | Fluoride toothpaste (1450 ppm) without zinc | Metataxonomics (16S rRNA); metatranscriptomics; KEGG pathways; species abundance; functional gene expression | Zinc toothpaste significantly altered microbiome composition and function vs control; increased Veillonella spp. and reduced Fusobacterium; reduced glycolysis and sugar metabolism pathways; increased lysine biosynthesis and nitrate reduction pathways | No clinical caries outcomes; short duration (6 weeks); industry-funded; adult population only; pooled transcriptomic samples; limited clinical correlation |
| Gugnani N; Gugnani S | 2025 | India | Commentary on systematic review and meta-analysis | Narrative commentary | Not applicable (secondary synthesis of clinical and in situ studies) | Not applicable (summarises mixed populations across included studies) | n=18 included studies (clinical trials + in situ studies) | Hydroxyapatite (various formulations: toothpaste, gel, mouthwash) | Not applicable (summary of multiple study designs and interventions) | HAP vs placebo; HAP vs fluoride; no intervention (as per included studies) | Caries incidence (dmft/DMFT; ICDAS); remineralization (%); lesion depth (µm); bacterial load (%) | HAP significantly better than placebo (pooled OR 2.51); non-significant difference vs fluoride (OR 1.1); in situ studies favoured HAP for lesion depth (−1.15 µm) and bacterial load (−65%); remineralization improvements non-significant | Commentary article (not primary data); dependent on included study quality; heterogeneity in study designs/outcomes; limited number of high-quality RCTs; potential interpretation bias |
| Anwar MA; Sayed GA; Hal DM; Hafeez MSAE; Shatat AS; Salman A; Eisa NM; Ramadan A; El-Shiekh RA; Hatem S; Aly SH | 2025 | Egypt | Narrative comprehensive review (multi-database literature synthesis) | Narrative review | Mixed (in vitro, in vivo, clinical studies included) | Not applicable (broad oral health conditions across populations) | n=358 included studies (from 500 screened) | Multiple herbal compounds (e.g. clove, aloe vera, peppermint, propolis, tea, neem, turmeric, garlic, polyphenols, flavonoids) | Multiple delivery forms (toothpaste, mouthwash, gels, extracts, chewing gum, hydrogels) | Not applicable (broad comparisons vs conventional agents e.g. fluoride, chlorhexidine, placebo) | Antimicrobial activity; anti-inflammatory markers; antioxidant capacity; plaque indices; gingival indices; biofilm formation; surrogate and clinical outcomes | Herbal agents demonstrated antimicrobial, anti-inflammatory, and antioxidant effects; multiple extracts reduced plaque, gingival inflammation, and cariogenic bacteria; some clinical trials showed comparable efficacy to chlorhexidine and improvements in oral health indices | Highly heterogeneous evidence base; narrative synthesis (no meta-analysis); variable study quality; mixed populations and outcomes; limited standardisation of formulations/doses; reliance on surrogate endpoints |
| Wierichs RJ; Kuruparan M; Ruthiraswaran A; Meyer-Lueckel H; Carvalho TS; Niemeyer SH | 2024 | Switzerland | In vitro experimental study (pH-cycling model) | Laboratory study | In vitro (controlled pH-cycling, biofilm-free model) | Bovine dentin specimens (sound and artificially demineralised) | n=187 specimens across 11 groups | Fluoride (0, 500, 1,100 ppm NaF); grape seed extract (GSE); (nano-)hydroxyapatite (nHA/HA); melaleuca oil (MO); propolis + myrrh; curcuma/clove extracts | Dentifrice slurries (1:3 with deionised water), applied via simulated brushing (2× daily) | Fluoride-free control (0 ppm NaF) and comparison across fluoride concentrations | Integrated mineral loss (ΔΔZ); lesion depth (ΔLD); mineral density profiles (TMR analysis) | Strong fluoride dose-response observed (r=0.681-0.861); NaF1100 most effective; fluoride-free agents showed limited benefit-GSE and MO reduced progression in demineralised dentin but all fluoride-free groups showed surface loss; nHA/HA not significantly different from control | Highly controlled in vitro model (no biofilm/saliva); bovine dentin (not human); mild demineralisation model (low caries risk simulation); surrogate outcomes; limited clinical generalisability |
| Maldupa I; Narbutaite J; Stanceviciene E; Viduskalne I; Kalnina J; Kronina L; Brinkmane A; Senakola E; Uribe SE | 2024 | Latvia & Lithuania | Cross-sectional survey study | Observational study | Community-based (household/family survey; schools) | Families (all ages: preschoolers, children, adolescents, adults) | n=1309 families; 5436 individuals | Fluoride exposure via toothpaste (categorised: none, <1000 ppm, 1000-1399 ppm, 1400-1500 ppm) | Self-reported toothpaste use with photographic verification of brands | Comparison across fluoride concentration categories and age groups | Prevalence of fluoride vs non-fluoride toothpaste use; distribution by age, region, and household | 15% used non-fluoride toothpaste; 12% used <1000 ppm; only 58% used optimal fluoride (≥1400 ppm); children most likely to use suboptimal/non-fluoride products; significant public health gap in fluoride use, especially in younger age groups | Cross-sectional design (no causality); self-reported data; differing data collection methods (online vs in-person); potential misclassification from product identification; no behavioural/clinical outcomes measured |
| Vajrabhaya LO; Benjasupattananan S; Sappayatosok K; Dechosilpa V; Korsuwannawong S; Sirikururat P | 2024 | Thailand | Randomised double-blind parallel-group clinical trial | Clinical (interventional) | In vivo (periodontitis patients undergoing NSPT) | Adults (20-70 years) with periodontitis (≥4 teeth with PD >4 mm; >30% BOP; >40% plaque) | n=54 (test n=22; active control n=13; benchmark n=19) | Aloe vera-based herbal formulation (multi-herbal extract blend) | Toothpaste; twice-daily brushing with oral hygiene instruction during scaling and root planing | Sodium bicarbonate toothpaste (active control); standard toothpaste (benchmark) | Plaque score (PS); bleeding on probing (BOP); probing depth (PD); clinical attachment level (CAL) | All groups showed reductions in PS, BOP, and PD; herbal toothpaste showed greater CAL reduction vs controls; significant BOP reduction at 4 weeks; no significant PS differences between groups | Small sample size; short follow-up (12 weeks); adjunct to NSPT (confounding treatment effect); periodontal outcomes only (no caries data); multi-ingredient formulation limits attribution |
| Nandhini G; Samraj JS; Anish SM; Ramachandran AK; Nirmala C; Denis GFL | 2024 | India | In vitro experimental study (pH-cycling model; comparative groups) | Experimental study | In vitro | Human premolar enamel specimens with artificial white spot lesions | n=24 (4 groups, n=6 each) | Strontium-doped bioactive glass; BioMin; NovaMin (with CHX pretreatment) | Dentifrice/application via brushing in pH-cycling model (14 days; twice daily) | Artificial saliva control; comparison across bioactive glass formulations | Energy-dispersive X-ray analysis (EDAX); calcium/phosphate ratio; mineral gain | All agents showed remineralization potential; strontium-doped bioactive glass + CHX showed greatest mineral gain; BioMin > NovaMin; fluoride-containing systems showed synergistic effect | Small sample size; in vitro model; artificial lesions; CHX pretreatment confounding effect; short duration; surrogate outcomes only; limited clinical generalisability |
| Duggal S; Chandrika PS; Nasyam FA; Singh DK; Devraj IM; Anand C | 2024 | India | Narrative comprehensive review (literature overview) | Narrative review | Not applicable (mixed evidence: in vitro, clinical, traditional use) | Not applicable (broad oral health applications across populations) | Not specified (non-systematic review) | Multiple herbal agents (e.g. neem, clove, tea tree oil, aloe vera, sage, chamomile, calendula, peppermint) | Multiple delivery forms (toothpaste, mouthwash, gels, oils, extracts) | Not applicable (broad comparisons vs conventional dental products) | Antimicrobial activity; anti-inflammatory effects; analgesic effects; plaque control; gingival health; surrogate and clinical outcomes | Herbal agents demonstrate antimicrobial, anti-inflammatory, and analgesic properties; potential benefits in plaque reduction, gingival health, and oral symptom management; increasing interest in holistic and natural oral care approaches | Lack of robust clinical evidence; limited high-quality trials; heterogeneity of formulations; poor standardisation; reliance on traditional/experimental data; potential safety and dosing concerns |
| Sanchez-Tito M; Tay LY; Zea-Gamboa F; Cartagena-Cutipa R; Flores-Gomez A; Spigno-Paco B; Cadenas TC; Diaz IE | 2024 | Peru | In vitro experimental study (antibacterial assays: disk diffusion, microdilution, agar well diffusion) | Experimental study | In vitro | Bacterial consortium (Streptococcus mutans, S. sanguinis, S. salivarius) | Repeated measures (n=35 total toothpaste tests; triplicate assays for EO testing) | Hypericum laricifolium essential oil (0.28% v/v in toothpaste) | Experimental toothpaste formulation; compared with six commercial toothpastes | Commercial toothpastes (e.g. Colgate Total, Colgate Herbal, Kolynos Herbal, Oral B); 0.12% chlorhexidine control | Bacterial growth inhibition zones (mm); MBC; CFU counts | Essential oil showed strong antibacterial activity (greater than CHX for some strains); experimental toothpaste demonstrated significant inhibition and similar performance to commercial toothpastes against bacterial consortium | In vitro design; no biofilm complexity beyond limited consortium; short-term outcomes; no remineralization or clinical caries data; formulation variability; no in vivo validation |
| Pawinska M; Paszynska E; Amaechi BT; Meyer F; Enax J; Limeback H | 2024 | Poland | Systematic review and meta-analysis (RCTs, in vivo and in situ clinical trials) | Systematic review & meta-analysis | Clinical (in vivo) + in situ | Humans (children and adults across included studies) | 18 studies identified; 5 clinical trials + 8 in situ studies included in meta-analysis | Hydroxyapatite (HAP; micro- and nano-forms) in oral care products (toothpaste, mouthwash, gel) | Various delivery formats (toothpaste, mouthwash, gel) across included studies | Placebo, no intervention, or fluoride-containing products (active controls) | Caries incidence (DMFT/DMFS, ICDAS); remineralization (mineral gain/loss %); lesion depth; bacterial load/adhesion | HAP demonstrated effectiveness in reducing caries risk; non-inferior to fluoride in clinical trials; modest remineralization benefit; significant reduction in lesion depth and bacterial adhesion (~65% reduction vs control); overall supports HAP as fluoride alternative | Heterogeneity of included studies; variability in formulations and concentrations; limited number of high-quality RCTs; reliance on proxy outcomes in in situ studies; some risk of bias; meta-analysis limited by data reporting variability |
| Latifi-Xhemajli B | 2024 | Kosovo / USA | Narrative literature review | Narrative review | Not applicable (mixed evidence: clinical, in vitro, observational studies) | Not applicable (summarises children and adults across included studies) | n=8 included studies (toothpaste-specific) | Xylitol (often in combination with fluoride or other agents) | Toothpaste (various formulations; sometimes combined with triclosan, fluoride, probiotics, erythritol) | Fluoride toothpaste; placebo; other xylitol-containing or combination products | Caries incidence (DMFT/DFS); Streptococcus mutans levels; Lactobacillus levels; remineralization; demineralization; plaque indices | Mixed findings: some studies show reduced caries increment and bacterial levels with xylitol-containing toothpaste; others show no additional benefit over fluoride; evidence inconsistent and limited for toothpaste-specific effects | Very limited number of toothpaste-specific studies; heterogeneity in formulations and study designs; conflicting results; reliance on combination products; lack of strong clinical evidence; conclusions largely narrative |
| Dumitrel SI; Matichescu A; Dinu S; Buzatu R; Popovici R; Dinu DC; Bratu DC | 2024 | Romania | Narrative comprehensive review (broad synthetic compounds in dentistry) | Narrative review | Not applicable (mixed: in vitro, clinical, in vivo studies synthesised) | Not applicable (broad dental populations and conditions) | Not specified (non-systematic synthesis) | Multiple synthetic compounds (e.g. chlorhexidine, octenidine, cetylpyridinium chloride, povidone-iodine, sodium hypochlorite, hydrogen peroxide) | Multiple delivery forms (mouthwash, toothpaste, gels, irrigants, varnishes, sprays, bleaching agents) | Not applicable (broad comparisons across compounds and clinical uses) | Plaque index; bleeding index; microbial counts; biofilm formation; clinical periodontal indices; disinfection efficacy; whitening outcomes | Synthetic compounds demonstrate strong antimicrobial, anti-plaque, and therapeutic effects across dental applications; e.g. CHX significantly reduces plaque and bleeding indices; OCT and CPC reduce bacterial load; NaOCl effective in endodontic disinfection; H₂O₂ effective for bleaching | Largely narrative synthesis; heterogeneous evidence base; limited critical appraisal; broad scope reduces specificity; potential bias in study selection; limited direct comparison between agents |
| Bass T; Hill CM; Cully JL; Li SR; Chi DL | 2024 | USA | Cross-sectional survey of medical providers (physicians, NPs, PAs) | Quantitative (cross-sectional survey) | Primary care settings (paediatric and family medicine) | Paediatric and family medicine providers treating children <18 years | n=354 | Fluoride (multiple modalities: topical fluoride, fluoride supplements, OTC fluoride toothpaste, fluoridated water) | Clinical application, prescribing, and recommendation practices (e.g. topical varnish, supplements, toothpaste advice) | Not applicable (comparative analysis between provider types rather than intervention vs control) | Provider beliefs (effectiveness of fluoride modalities); clinical practices (application/prescription/recommendation rates); experiences with fluoride hesitancy; barriers to oral health integration | Most providers viewed fluoride as effective (75.7% very effective overall; 61.9% for fluoridated water); 87.3% recommend fluoride toothpaste but only 44.1% apply topical fluoride and 30.8% prescribe supplements; fluoride hesitancy perceived as a minor issue (82.5% small/not a problem); key barrier was lack of time (58.8%), especially in family medicine (65.6% vs 50.3%) | Convenience sampling limits generalisability; predominantly white physician sample; underrepresentation of NPs/PAs; self-reported data; limited geographic scope |
| Gomersall JC; Slack-Smith L; Kilpatrick N; Muthu MS; Riggs E | 2024 | Australia, United Kingdom, India | Systematic review of RCTs (Cochrane Review) | Quantitative (systematic review and meta-analysis of RCTs) | Community, primary care, and maternal-child health settings | Pregnant women, new mothers, and primary caregivers of infants (<12 months), with child outcomes up to 6 years | n=23,732 caregivers across 17 RCTs | Multiple: oral health education (diet/feeding advice, breastfeeding support, oral hygiene), antimicrobial agents (chlorhexidine, iodine-NaF), xylitol | Behavioural interventions (education/promotion), clinical maternal interventions (antimicrobial treatments, xylitol use) | Standard care, placebo, or alternative interventions | Caries outcomes (presence, dmfs, dmB); child oral health behaviours; maternal behaviours and microbiological outcomes | Diet and feeding advice probably reduces ECC risk (~15% reduction; RR 0.85); breastfeeding support shows little/no effect; oral hygiene + diet advice shows little/no clear effect; antimicrobial maternal treatments show uncertain benefit; xylitol may reduce dmB but evidence limited; overall evidence mostly low-very low certainty except diet/feeding advice (moderate certainty) | Substantial heterogeneity in interventions and outcomes; many studies with unclear/high risk of bias; small sample sizes in comparisons; limited number of trials per outcome; low-very low certainty for most findings; inconsistent reporting |
| Folayan MO; Olagunju MT; Abodunrin OR; Alade OT | 2024 | Nigeria, China | Scoping review (JBI methodology; PRISMA-ScR reported) | Mixed (descriptive synthesis of experimental studies and ethnobotanical surveys) | Community, traditional healer settings, laboratory (for experimental studies) | Not applicable (included studies: ethnobotanical surveys and experimental lab-based studies on plant extracts) | n=12 included studies (from 584 records) | Plant-based traditional medicines (roots, leaves, bark, seeds, whole plants; occasional mineral adjuncts) | Topical applications, mouth rinses, gargling, chewing; limited systemic use (inhalation, ingestion) | Not applicable (scoping review; no unified comparator) | Oral diseases managed (caries, periodontal disease, ulcers, abscesses, halitosis, oral infections); antimicrobial activity against oral pathogens; ethnobotanical usage patterns | All included studies utilised plant-based remedies; majority were ethnobotanical surveys; experimental studies showed plant extracts had antimicrobial activity against cariogenic (e.g. S. mutans, S. sobrinus) and periodontal pathogens; wide range of oral conditions managed including caries, gingivitis, ulcers, and halitosis; administration predominantly topical (mouth rinses, chewing, application); significant heterogeneity in plant species (29-62 per study) and families (15-29) | Very limited number of studies relative to continent (only 12 studies across 8 countries); exclusion of non-English literature; predominance of descriptive ethnobotanical data with limited clinical evidence; lack of robust clinical trials assessing safety and efficacy; heterogeneity in methods and outcomes limits synthesis; potential toxicity and safety concerns not well evaluated |
| Srisomboon S; Intharah T; Jarujareet U; Toneluck A; Panpisut P | 2024 | Thailand | In vitro experimental study | Laboratory-based experimental study (quantitative) | Laboratory (biomaterials / dental research setting) | Demineralised human dentine specimens | n=48 teeth (n=8 per group; 6 groups) | Propolis extract; Aloe vera extract (in artificial saliva substitutes) | Artificial saliva formulations with varying concentrations: 5% propolis, 5% aloe vera, 2.5% each, or none | Commercial saliva substitute (Biotene Oral Rinse) + deionised water control | Rheological properties (viscosity); dentine remineralisation (ATR-FTIR mineral-to-collagen ratio); SEM-EDX (surface mineral deposition, tubule occlusion) | Propolis-containing formulations (especially 5%) showed greatest remineralisation (median ~25.48% increase at 14 days); increased mineral precipitation and dentinal tubule occlusion observed on SEM; aloe vera showed minimal effect; all experimental formulations had lower viscosity than Biotene but within physiological saliva range; slight shear-thinning behaviour observed | In vitro design limits clinical applicability; short duration (14-day pH cycling); high variability between tooth specimens; lack of true clinical endpoints; composition of propolis not fully characterised; potential issues with stability and staining not assessed; no long-term biofilm model or in vivo validation |
| Karia S; Baerts E; Coventry H; Taylor G | 2024 | UK | Commentary on observational cohort study | Narrative commentary | Not applicable (secondary appraisal of clinical cohort study) | Infants/young children (1-30 months; high caries risk) | n=102 children (original cohort study) | Xylitol (25% toothpaste formulation) | Toothpaste used twice daily over 24 months (as per underlying study) | No control group in underlying study; pre-post comparison of same cohort | Streptococcus mutans levels (Dentocult SM); ICDAS caries assessment; prevalence change over time | 19.1% reduction in S. mutans prevalence after xylitol toothpaste use; statistically significant decrease (p=0.002); suggests potential anticariogenic effect but evidence considered weak and preliminary | Commentary (not primary data); underlying study lacked control group; small sample; high attrition; poor standardisation (diet, oral hygiene not controlled); unclear toothpaste composition; potential confounding factors; limited methodological rigour |
| Meyer F; Schulze Zur Wiesche E; Amaechi BT; Limeback H; Enax J | 2024 | Germany / USA / Canada | Narrative review (non-systematic literature synthesis) | Narrative review | Mixed (in vitro, in vivo, clinical evidence discussed) | Not applicable (broad populations across lifespan) | Not specified (non-systematic review) | Multiple agents: fluoride, hydroxyapatite, calcium phosphates, xylitol, polyphenols, chlorhexidine, zinc/stannous salts | Multiple delivery forms (toothpaste, mouthwash, gels, varnishes; mechanical and chemical plaque control strategies) | Not applicable (broad comparisons across preventive strategies) | Caries etiology (plaque, sugar, pH); remineralization/demineralization processes; plaque control; antimicrobial effects; preventive strategies | Hydroxyapatite described as versatile non-fluoride agent with multiple mechanisms (remineralization, pH buffering, plaque reduction) and clinical non-inferiority to fluoride; caries prevention requires combined approach (plaque control + sugar reduction + remineralization); fluoride effective but with limitations (surface-level effect, toxicity concerns at high exposure) | Narrative, non-systematic methodology; potential industry bias (author affiliations); broad scope limits critical appraisal; heterogeneous evidence base; selective emphasis on biomimetic agents; no formal risk of bias assessment |
| do Amaral Silva M; Valadas LAR; Lopes de Oliveira GA; Rodrigues Neto EM; de Alencar Junior EA; Dantas Lobo PL; Dantas TCFB; Bandeira MAM; de Franca Fonteles MM; Baptista GR | 2024 | Brazil | Randomized, double-blind, longitudinal clinical trial (parallel groups) | Quantitative (RCT) | Clinical (orthodontic patients; in vivo) | Adolescents (12-18 years) with fixed orthodontic appliances, caries-free (ICDAS II = 0) | n=76 (38 per group) | Brazilian red propolis (1%) + fluoride (1500 ppm MFP) | Toothpaste; 3× daily brushing for 28 days (standardised oral hygiene protocol) | Fluoridated toothpaste (1500 ppm MFP) | Salivary biomarkers (pH, total protein, amylase, IL-10); salivary flow; plaque index | No significant changes in pH, total protein, amylase, or salivary flow; significant reduction in IL-10 in both groups (greater in propolis group); plaque index significantly reduced, with greater reduction in propolis group (P <0.0001 vs 0.03) | Short follow-up (28 days); narrow age range; single-centre study; orthodontic population limits generalisability; no long-term clinical caries outcomes; potential behavioural confounding (oral hygiene standardisation) |
| Rajendran R; Antony S DP; Ashik P M; Bharath S; Thomas AJ; Heboyan A | 2024 | India | Randomised controlled trial (parallel 3-arm clinical trial) | Clinical (interventional) | In vivo (post-orthodontic patients with WSLs) | Adolescents and young adults (15-25 years) with white spot lesions post-orthodontic debonding | n=90 (30 per group) | Strontium-doped nano-hydroxyapatite (SrnHAp); CPP-ACP | Topical paste/cream applied twice daily for 6 weeks (with standard dentifrice use) | Regular dentifrice (control); CPP-ACP cream (active comparator) | Visual WSL scoring (Ekstrand criteria); lesion severity scores (pre/post); intergroup comparisons | SrnHAp and CPP-ACP significantly improved remineralisation vs control (p<0.001); SrnHAp showed greater remineralisation than CPP-ACP (higher proportion score 0 post-treatment); control showed no improvement | Short follow-up (6 weeks); visual scoring only (no objective mineral quantification); single-centre; no long-term caries outcomes; potential compliance variability; limited blinding detail |
| Barahuie F; Dianat T; Ghaderi Nejad N; Shahbakhsh M; Kordi Tamandani DM | 2023 | Iran | Experimental laboratory formulation and antimicrobial evaluation study | Experimental study | In vitro (product formulation and microbiological testing) | Not applicable (laboratory-based bacterial assays; no human participants) | Not specified (multiple product samples; microbial assays conducted in duplicate/standardised testing) | Salvadora persica extract; Moringa oleifera extract (combined herbal formulation) | Toothpaste, mouthwash, and chewing gum formulations (organic/herbal products) | Not explicitly defined control; comparison to standard allowable microbial limits and antibacterial reduction thresholds | Microbial count (CFU); antibacterial activity against Pseudomonas aeruginosa, Escherichia coli, Staphylococcus aureus, Enterococcus hira; physicochemical properties (moisture, ash, heavy metals) | Herbal formulations met acceptable microbial standards; demonstrated strong antibacterial activity (≥10⁵ reduction in bacterial counts across tested strains); products considered stable and within quality limits | No clinical or in vivo validation; no comparison to conventional fluoride products; lack of standardised control group; surrogate microbiological outcomes only; unclear sample replication and statistical analysis |
| Patel MK; Milano M; Messer RL | 2023 | USA | Cross-sectional survey study (questionnaire-based) | Observational study | Community (private practice paediatric dentists; online survey) | Paediatric dentists (southeastern and western USA; private practice) | n=205 responses (from 6490 invited; 3.1% response rate) | Fluoride-free remineralising agents (e.g. nano-hydroxyapatite, theobromine, tricalcium phosphate, CPP-ACP, arginine, xylitol-based systems) | Not applicable (survey of awareness, attitudes, and clinical recommendations) | Comparisons by region (western vs southeastern), age, and training background | Awareness of fluoride-free agents; recommendation behaviour; frequency of fluoride refusal; brand recognition; attitudes to fluoride alternatives | Moderate awareness reported (~55.8%), but 58.4% could not recognise any fluoride-free brands; 30.4% would recommend fluoride-free toothpaste to all patients; majority would recommend alternatives only for fluoride-averse patients; western-trained dentists more likely to recommend fluoride-free options; “lack of research” most cited barrier (48.9%) | Low response rate (3.1%); responder bias; limited to two US regions; self-reported data; not generalisable; awareness ≠ clinical competence; no clinical outcomes assessed |
| Latifi-Xhemajli B; Kutllovci T; Begzati A; Rexhepi A; Ahmeti D | 2023 | Kosovo | Prospective longitudinal cohort study (24-month follow-up) | Observational study (longitudinal) | Clinical (in vivo; community-based preventive intervention) | Infants/young children (baseline mean age 6.7 months; high caries risk; mother-infant dyads) | n=102 (from initial 270; significant attrition) | Xylitol (25% toothpaste formulation) | Toothpaste; twice-daily brushing over 24 months (home-based intervention) | No control group (pre-post comparison within cohort) | Mutans streptococci (SM) prevalence (Dentocult CFU categories); ICDAS caries assessment at baseline | Significant reduction in SM: bacteria-free increased 27.4%→44.5%; high SM (≥10⁵ CFU/ml) reduced 17.1%→4.9%; overall SM prevalence decreased; suggests antibacterial effect and potential ECC prevention benefit | No control group; high attrition (270→102); observational design (no causality); potential Hawthorne/compliance effects; surrogate microbiological outcomes; limited direct caries outcome data; generalisability limited to high-risk population |
| Paszynska E; Pawinska M; Enax J; Meyer F; Schulze Zur Wiesche E; May TW; Amaechi BT; Limeback H; Hernik A; Otulakowska-Skrzynska J; Krahel A; Kaminska I; Lapinska-Antonczuk J; Stokowska E; Gawriolek M | 2023 | Poland | Double-blinded randomized controlled trial (non-inferiority; 18 months; parallel-group) | Randomized controlled trial (RCT) | Clinical (in vivo; adult population) | Adults (18-45 years; generally healthy; ≥10 caries-free molars/premolars at baseline) | ITT n=189; PP n=171 | Hydroxyapatite (10% fluoride-free toothpaste) | Toothpaste; twice-daily brushing (3 min) with electric toothbrush over 18 months | Sodium fluoride toothpaste (1450 ppm fluoride; active control) | Primary: DMFS index (no increase); Secondary: DIAGNOcam caries lesions, Plaque Control Record (PCR) | Hydroxyapatite toothpaste non-inferior to fluoride: ~89.3% (HAP) vs 87.4% (fluoride) showed no DMFS increase; minimal DMFS change; similar caries lesion progression; no significant differences in plaque reduction; overall equivalence suggested | Adult population (limited generalisability to children); relatively low caries-risk cohort; no placebo group; clinical setting with regular follow-up; diet not controlled; reliance on DMFS and DIAGNOcam rather than radiographs |
| Rajendran R; Antony DP | 2023 | India | In vitro experimental laboratory study (development + remineralization assessment) | Laboratory study (in vitro) | Laboratory (extracted human enamel specimens) | Extracted human premolars (sound enamel; orthodontic extractions) | n=30 enamel specimens | Strontium-doped nano-hydroxyapatite (Sr-nHAp) paste (lab-developed; ~50% Sr-nHAp dentifrice formulation) | Paste/dentifrice; applied via brushing twice daily for 3 minutes over 28 days (simulated oral conditions with artificial saliva) | No active comparator (within-sample comparison: sound vs demineralised vs remineralised enamel) | Primary: Calcium and phosphorus content (SEM-EDAX); Secondary: Surface morphology (SEM) | Significant increase in calcium and phosphorus after remineralisation compared to demineralised enamel (p=0.001); values returned close to baseline sound enamel; SEM showed smoother surfaces and mineral deposition → strong remineralisation potential | In vitro design (limited clinical applicability); no comparator group; short duration; small sample size; does not replicate full oral environment; requires in vivo validation |
| Ahmed OAK; Sibuyi NRS; Fadaka AO; Maboza E; Olivier A; Madiehe AM; Meyer M; Geerts G | 2023 | South Africa | In vitro experimental laboratory study (antimicrobial + cytotoxicity evaluation of nanoparticle toothpaste formulation) | Laboratory study (in vitro) | Laboratory (oral microbial cultures and human buccal mucosa fibroblast cells) | Oral microbes (S. mutans, S. sanguinis, L. acidophilus, C. albicans) + human buccal mucosa fibroblast (BMF) cells | Not stated (triplicate experiments; multiple assays) | Gum arabic-silver nanoparticles (GA-AgNPs) incorporated into toothpaste (GA-AgNPs_TP-1) | Nanoparticle toothpaste formulation (AgNPs mixed with low-activity commercial toothpaste; exposure assays up to 24 h) | Commercial toothpastes (TP1-4); TP-1 alone; GA-AgNPs alone | Primary: Antimicrobial activity (ZOI, MIC, CFU); Secondary: Cytotoxicity (MTT assay), time-concentration response | GA-AgNPs retained antimicrobial activity after incorporation into toothpaste; significant inhibition of oral microbes (dose-dependent); TP alone inactive at test concentration; rapid antimicrobial effect (<1 h) but also dose- and time-dependent cytotoxicity to BMF cells | In vitro design; cytotoxicity to human cells; non-selective antimicrobial effects; short exposure models vs real use; no clinical validation; potential nanoparticle toxicity concerns |
| Amanpour S; Akbari Javar M; Sarhadinejad Z; Doustmohammadi M; Moghadari M; Sarhadynejad Z | 2023 | Iran | Systematic review (PRISMA-guided synthesis of clinical and in vitro studies on herbal oral health interventions) | Systematic review (no meta-analysis; qualitative synthesis) | Various (clinical and laboratory settings across included studies) | Patients with oral diseases (gingivitis, periodontitis, aphthous ulcers, oral lichen planus, candidiasis, etc.) | 49 studies included; total n ≈ 2,909 participants across studies | Multiple herbal compounds (e.g., chamomile, Aloe vera, green tea, neem, triphala, myrrh, sage, etc.) | Various formulations (gels, mouth rinses, pastes, capsules, oils, etc.) | Varied comparators (placebo, conventional treatments, chlorhexidine, fluoride products, etc.) | Gingival index, plaque index, periodontal outcomes, pain scores (VAS), lesion healing, quality of life | Overall, most studies reported statistically significant improvements in gingival, plaque, and symptom outcomes; herbal products generally demonstrated effectiveness and favourable safety profiles, though heterogeneity was high | Heterogeneous study designs and outcomes; many studies with unclear/high risk of bias; small sample sizes and short durations; limited standardisation; lack of robust clinical evidence and need for higher-quality trials |
| Pushpalatha C; Gayathri VS; Sowmya SV; Augustine D; Alamoudi A; Zidane B; Hassan Mohammad Albar N; Bhandi S | 2023 | India, Saudi Arabia, United States | Narrative review (comprehensive literature overview of nanohydroxyapatite applications in dentistry) | Narrative review (no formal systematic methodology or meta-analysis reported) | Various (preventive, restorative, implantology, laboratory and clinical contexts across included studies) | Not applicable (review of multiple study populations including in vitro, animal, and clinical studies) | Not applicable (synthesis of multiple studies rather than a single sample) | Nanohydroxyapatite (nHA) (including doped/modified forms and composites) | Multiple formulations (dentifrices, mouth rinses, gels, pastes, coatings, composites, scaffolds, GIC additives, etc.) | Varied comparators (fluoride, CPP-ACP, bioactive glass, conventional materials, placebo, etc.) | Remineralisation, dentine hypersensitivity, implant osseointegration, microhardness, antibacterial activity, bond strength, tissue regeneration outcomes | nHA demonstrates strong remineralisation potential, biocompatibility, and versatility across applications (caries prevention, hypersensitivity, implantology, regeneration), with performance often comparable to or synergistic with fluoride, though evidence varies by context | Lack of robust long-term clinical evidence; heterogeneity of included studies; concerns regarding cytotoxicity and safety require further investigation; need for standardised, high-quality trials |
| Shalini S; Sharma S; Anand A; Almalki SA; Biswas A; Sharma M; Sihag T; Ojha A; Garg Y; Paiwal K | 2023 | India | Cross-sectional observational study (questionnaire + clinical examination) | Observational study (cross-sectional) | Clinical (in vivo; school-based oral health assessment) | Children with special healthcare needs (aged 4-15 years; special schools) | n=124 | Not applicable (no intervention; epidemiological study) | Not applicable (oral health assessment; no product/formulation tested) | None (descriptive study; no comparator group) | Dental caries prevalence (%); deft index; oral hygiene status; oral hygiene habits (brushing frequency, fluoride use, flossing) | High caries prevalence (65%); poor oral hygiene (75%); mean deft 2.8; high non-fluoride use (70%); suboptimal oral hygiene practices common in this population | Cross-sectional design (no causality); convenience sampling; limited geographic generalisability; self-reported habits; no intervention or comparative analysis |
| Gloag ES; Khosravi Y; Masters JG; Wozniak DJ; Amorin Daep C; Stoodley P | 2023 | United States, United Kingdom | In vitro experimental laboratory study (biofilm mechanics and biophysical analysis) | Laboratory study (in vitro) | Laboratory (mono-species and saliva-plaque biofilm models; rheology and microscopy) | Streptococcus gordonii biofilms; saliva-plaque biofilms (healthy donor-derived) | n=3 biological replicates (duplicate biofilms) for rheology; n=4 biological replicates (triplicate) for indentation | Dual Zinc + Arginine (0.96% zinc ions [zinc oxide + zinc citrate] + 1.5% L-arginine) | Dentifrice formulation and solution exposure (2-minute treatment; biofilm assays) | Zinc alone; arginine alone; PBS control; stannous fluoride toothpaste (for paste comparison) | Biofilm mechanical properties (torque, shear stress, Young’s modulus); EPS volume (CLSM/COMSTAT); biofilm detachment; rheological behaviour | DZA significantly reduced biofilm mechanical integrity (lower torque, lower Young’s modulus); increased susceptibility to shear removal; reduced EPS volume and increased heterogeneity; effects greater than zinc or arginine alone (additive interaction); DZA dentifrice more effective than stannous fluoride in weakening biofilms | In vitro models (limited clinical translation); short exposure time (2 min); healthy biofilm models (non-cariogenic); industry funding/conflict of interest; complex biophysical endpoints not directly linked to clinical outcomes |
| Rosenauer T; Basche S; Flemming J; Hannig C; Konig B; Hannig M | 2023 | Germany | In situ clinical-experimental pilot study | In situ study (clinical experimental) | Oral environment (intraoral splint model with enamel slabs worn overnight) | Healthy adult volunteers (good oral hygiene; no active disease) | n=8 subjects (6 female, 2 male; multiple enamel samples per subject) | Fluoridated dentifrices (1400 ppm AmF; AmF + SnF2) and fluoride-free hydroxyapatite dentifrice (HAP) | Toothpaste use (2-day wash-in per product; direct and indirect application regimens; overnight 8 h exposure) | No toothpaste control (brushing without dentifrice) | Initial bacterial colonisation (cells/cm² via fluorescence microscopy); bacterial viability (BacLight); glucan formation (ConA staining); pellicle ultrastructure (TEM) | No statistically significant differences in initial bacterial colonisation, viability, glucan formation, or pellicle structure between dentifrices (fluoride or HAP) and control; no difference between direct vs indirect application; dentifrices did not reduce early biofilm formation in good oral hygiene subjects | Small sample size (pilot study); short-term single application (8 h overnight); healthy participants with low baseline plaque; in situ model limitations (not fully in vivo); potential variability in biofilm distribution; not powered to detect small differences |
| Ahmed O; Sibuyi NRS; Fadaka AO; Madiehe MA; Maboza E; Meyer M; Geerts G | 2022 | South Africa | Narrative review | Review (nanotechnology / dental therapy) | Not applicable (literature synthesis across dental applications) | Not applicable (no primary population) | Not applicable | Silver nanoparticles (AgNPs), particularly plant-extract synthesised (green synthesis; phytochemical-mediated) | Various potential dental applications (toothpaste, varnish, composites, irrigants, coatings, mouth rinses) | Not applicable | Antimicrobial activity (MIC, MBC vs oral pathogens); antibiofilm effects; remineralisation potential; clinical outcomes (e.g., caries arrest, biofilm reduction); biocompatibility and toxicity profiles | AgNPs show strong broad-spectrum antimicrobial activity against oral pathogens (e.g., S. mutans) with lower MIC than other nanoparticles; can be incorporated into dental materials without compromising properties; clinical studies suggest nano-silver fluoride (NSSF) comparable to SDF but without staining and at lower cost; plant-synthesised AgNPs offer environmentally friendly, potentially biocompatible alternatives with similar or enhanced antimicrobial effects | Narrative review (not systematic); heterogeneity in synthesis methods, particle size, and formulations; limited high-quality clinical trials (many in vitro); potential toxicity concerns (dose-, size-, and surface-dependent); long-term safety and standardisation unclear; possible environmental and biological accumulation risks |
| Palka L; Nowakowska-Toporowska A; Dalewski B | 2022 | Poland | Narrative review (literature synthesis on chlorhexidine effects and adverse outcomes) | Narrative review | Not applicable (mixed: in vitro, in vivo, clinical and observational studies synthesised) | Not applicable (broad dental and medical populations) | Not applicable | Chlorhexidine (CHX) | Multiple delivery forms (mouthwash, gels, toothpastes, irrigants) | Not applicable | Antimicrobial efficacy; plaque inhibition; oral microbiome changes; systemic effects (blood pressure, metabolic outcomes); cytotoxicity; resistance development | CHX effective broad-spectrum antiseptic but associated with microbiome disruption, increased blood pressure, altered salivary metabolites, potential cytotoxicity to fibroblasts/osteoblasts, and risk of antimicrobial resistance and cross-resistance; prolonged or excessive use linked to adverse systemic and oral effects | Narrative design (no systematic methodology); heterogeneous evidence base; limited high-quality clinical data for some outcomes; potential publication bias; reliance on secondary data; causality not established for many associations |
| ALHumaid J; Bamashmous M | 2022 | Saudi Arabia | Systematic review and meta-analysis (PRISMA-guided; pooled quantitative synthesis) | Systematic review and meta-analysis | Mixed (clinical trials and observational human studies) | Children and adults (varied populations across included studies) | n=30 studies included (≥1-year follow-up; human studies) | Xylitol | Multiple delivery forms (chewing gum, candies, lozenges, toothpaste, mouth rinse, food products) | Non-xylitol controls (placebo, standard care, or alternative preventive measures) | Dental caries outcomes (DMFS/DMFT/dfs); caries incidence/prevented fraction | Xylitol products significantly reduced caries vs controls (SMD −0.099 fixed; −0.089 random); overall preventive fraction ~17%; most effective at 5-10 g/day, 3-5× daily; chewing gum and candies commonly studied | Heterogeneity high (I² up to 95.8%); limited number of high-quality trials; variability in product type, dose, and populations; inconsistent effect sizes; xylitol not studied as standalone intervention; moderate-low certainty overall |
| Yazdanian M; Rostamzadeh P; Rahbar M; Alam M; Abbasi K; Tahmasebi E; Tebyaniyan H; Ranjbar R; Seifalian A; Yazdanian A | 2022 | Iran | Narrative review (comprehensive literature review of green-synthesised metal nanoparticles in dentistry) | Narrative review | Not applicable (synthesis of in vitro, in vivo, and applied dental studies) | Not applicable (broad dental/biomedical applications across studies) | Not applicable | Green-synthesised metal nanoparticles (e.g., Ag, Au, Cu, ZnO, TiO₂; plant-mediated synthesis) | Multiple dental applications (toothpaste, mouthwash, restorative materials, implants, drug delivery systems) | Not applicable | Antimicrobial activity; antibiofilm effects; remineralisation; tissue regeneration; drug delivery; cytotoxicity/biocompatibility | Green-synthesised nanoparticles demonstrate strong antimicrobial and antibiofilm activity against oral pathogens (e.g., S. mutans), potential for remineralisation and tissue engineering, and wide applicability across dental materials; plant-mediated synthesis offers lower toxicity and environmentally friendly production with comparable or enhanced performance vs conventional methods | Narrative review (no systematic methodology); heterogeneity in nanoparticle types, synthesis methods, and applications; limited high-quality clinical evidence; concerns regarding nanotoxicity, long-term safety, and standardisation of synthesis and dosing remain |
| George JA; Srinivasan B; Kailasam V | 2022 | India | Randomized controlled trial (parallel-group, 2-arm; blinded outcome assessment) | Primary clinical study (RCT) | Single-centre clinical (orthodontic department; in vivo) | Orthodontic patients aged 13-30 years on fixed appliances (post-leveling/alignment, good oral hygiene) | n=34 (17 experimental, 17 control) | Active oxygen-containing toothpaste (Blue M) | Toothpaste use during routine oral hygiene (4-week intervention) | Fluoridated toothpaste (Colgate Total) | Streptococcus mutans counts (RT-PCR, Ct values); white spot lesions (ICDAS II, DIAGNOdent) | Active oxygen toothpaste showed slightly lower S. mutans levels vs fluoridated toothpaste, but not statistically significant (P=0.70); no meaningful change in white spot lesions in either group; both toothpastes showed comparable effectiveness | Small sample size; short follow-up (4 weeks); multifactorial confounding factors (diet, saliva, compliance); limited generalisability; lack of long-term outcomes |
| Salah R; Afifi RR; Kehela HA; Aly NM; Rashwan M; Hill RG | 2022 | Egypt | Randomized controlled trial (double-blind, 3-arm parallel design) | Primary clinical study (RCT) | Single-centre clinical (post-orthodontic patients; in vivo) | Post-orthodontic patients aged 14-26 years with ICDAS II score 2 white spot lesions | n=60 lesions (20 per group; Bio-BAG, N-BAG, CPP-ACP) | Bioactive glass 45S5 (BiominF, Novamin) | Combined in-office application (week 1) + home-use toothpaste/paste (4 weeks), with follow-up to 6 months | CPP-ACP (Recaldent) | White spot lesion size (digital image analysis, WL%); DIAGNOdent fluorescence readings | All groups showed significant WSL regression over 6 months; BiominF demonstrated significantly greater lesion reduction (~64.8%) compared to Novamin (~32.3%) and CPP-ACP (~31.7%); DIAGNOdent scores mirrored improvements, with greatest reduction in BiominF group | Single-centre study; compliance may influence outcomes; socioeconomic factors not assessed; limited external generalisability despite randomisation |
| Vaziriamjad S; Solgi M; Kamarehei F; Nouri F; Taheri M | 2022 | Iran | In vitro experimental study | Primary laboratory study | In vitro (Streptococcus mutans culture model) | Streptococcus mutans (ATCC 35668; no human participants) | Not applicable | l-arginine (5-100 µM concentrations) | Solution added to bacterial culture (growth, biofilm, and susceptibility assays) | Control (no arginine exposure) | Bacterial growth rate (optical density); biofilm formation (microtiter assay); antibiotic susceptibility (disk diffusion) | l-Arginine showed concentration-dependent effects: enhanced growth at 100 µM (non-significant); increased biofilm at low concentrations (5-10 µM) but reduced biofilm at higher concentrations (50-100 µM); decreased antibiotic susceptibility (reduced inhibition zones) for several antibiotics at higher concentrations | In vitro design limits clinical applicability; single-species model (no oral biofilm complexity); lack of in vivo validation; variability in concentration effects; findings may not translate directly to clinical caries prevention |
| Biria M; Rezvani Y; Roodgarian R; Rabbani A; Iranparvar P | 2022 | Iran | Double-blind randomized controlled trial (parallel-group) | Primary clinical study (RCT) | Clinical (in vivo; dental school setting) | Healthy dental students (18-30 years; routine oral hygiene; caries-free/low disease burden) | n=60 (30 per group) | Bamboo salt (herbal toothpaste; mineral-rich alkaline salt) | Toothpaste; twice-daily brushing (Bass technique) for 4 weeks | Conventional fluoridated toothpaste (Crest Complete®; 1450 ppm NaF) | Salivary Streptococcus mutans and Lactobacillus counts (log CFU/mL; baseline vs 4 weeks) | Both groups showed significant reduction in S. mutans and Lactobacillus (P<0.001); no significant difference between herbal and conventional toothpaste (P=0.530; P=0.137); comparable antibacterial efficacy | Dental student population (limited generalisability); short follow-up (4 weeks); compliance-dependent outcomes; potential unblinding due to taste; only antibacterial outcomes assessed (no caries endpoints) |
| Anil A; Ibraheem WI; Meshni AA; Preethanath RS; Anil S | 2022 | India / Saudi Arabia / Qatar | Scoping review (PRISMA-ScR guided; PCC framework) | Scoping review | Mixed (in vitro, in vivo, and clinical studies on dentifrices) | Human and laboratory-based studies (primary and permanent teeth; varied populations across included studies) | n=28 studies included (from 59 screened) | Nano-hydroxyapatite (nHAp) | Dentifrices (toothpaste formulations containing nHAp; various concentrations typically 1-25%, commonly ~10%) | Comparators varied (fluoride dentifrices, CPP-ACP, NovaMin, other non-fluoride agents) | Remineralisation of early caries; demineralisation inhibition; dentinal hypersensitivity; enamel surface changes; white spot lesions | nHAp dentifrices demonstrate remineralisation potential, caries inhibition, reduced demineralisation, and decreased dentinal hypersensitivity; optimal concentration ~10%; evidence suggests comparable or adjunctive role to fluoride, but insufficient high-quality evidence in primary teeth and long-term outcomes | Heterogeneity in study design, populations, concentrations, and outcomes; limited standardisation; inclusion of in vitro data limits clinical applicability; lack of long-term and paediatric-specific evidence; variability in protocols and formulations |
| Prince A; Roy S; McDonald D | 2022 | USA | In vitro experimental study | Primary laboratory study | In vitro (Streptococcus mutans culture; well diffusion and checkerboard assays) | Streptococcus mutans (ATCC 25175; no human participants) | Not applicable | Cranberry extracts (proanthocyanidin-rich; multiple types) + Manuka honey / methylglyoxal (MGO) | Solutions tested alone and in combination (pairwise antimicrobial assays) | Individual agents alone (cranberry extracts, Manuka honey, MGO; plus chlorhexidine and commercial mouthwashes as controls) | Zone of inhibition (agar diffusion); minimum inhibitory concentration (MIC); fractional inhibitory concentration (FIC index); biofilm inhibition potential | Synergistic antimicrobial effects observed for specific combinations (notably cranberry extracts Type R and RE with Manuka honey or MGO), showing significantly greater inhibition of S. mutans than individual agents; FIC indices <1 confirm synergy; combinations outperformed some commercial mouthwashes in vitro | In vitro design limits clinical applicability; single-species model (does not reflect complex oral biofilm); diffusion limitations in agar assays; variability in extract composition; no in vivo or clinical validation |
| Kumar R; Mirza MA; Naseef PP; Kuruniyan MS; Zakir F; Aggarwal G | 2022 | India / Saudi Arabia | Narrative review | Review article | Mixed (in vitro, in vivo, and clinical evidence across oral health applications) | Human, laboratory, and preclinical studies across multiple dental conditions (caries, gingivitis, periodontitis, oral cancer) | Not applicable | Natural products (essential oils, herbal extracts, phytoconstituents; e.g., neem, clove, curcumin, essential oils) ± nanotechnology-based delivery systems | Various delivery forms (toothpastes, mouthwashes, gels, nanoformulations such as liposomes, nanoemulsions, nanoparticles) | Comparators varied (conventional antimicrobials, fluoride products, chlorhexidine, synthetic agents) | Antimicrobial activity; biofilm inhibition; anti-inflammatory effects; remineralisation; anticancer effects; pharmacokinetic enhancement | Natural products demonstrate broad antimicrobial, anti-inflammatory, antioxidant, and anti-cariogenic properties; nanoformulations improve solubility, bioavailability, stability, and therapeutic efficacy; potential to overcome biofilm resistance and enhance oral health outcomes | Heterogeneity of included studies; reliance on in vitro/preclinical data; lack of standardisation; limited clinical evidence; variability in formulations and dosing; regulatory and quality control challenges |
| Poppolo Deus F; Ouanounou A | 2022 | Canada | Narrative review | Review article | Mixed (literature review across dental applications of chlorhexidine) | Human, clinical, and laboratory studies across oral health conditions (gingivitis, periodontitis, caries, oral surgery, implants) | Not applicable | Chlorhexidine (CHX) | Various formulations (mouthwash, gel, chips, varnish; primarily 0.12-0.2% mouthwash) | Comparators varied (mechanical plaque control, other antiseptics, gels, varnishes) | Plaque inhibition; gingivitis reduction; antimicrobial effects; periodontal outcomes; adverse effects | CHX is an effective adjunctive antimicrobial, particularly when mechanical cleaning is not possible; mouthwash (0.12-0.2%) is most effective formulation for short-term plaque and gingivitis control; CHX chips beneficial for sustained delivery in periodontal and peri-implant therapy; limited evidence for caries prevention | Heterogeneity of evidence; limited long-term clinical benefit beyond adjunctive use; adverse effects (tooth staining, taste disturbance, mucosal effects); concerns regarding antimicrobial resistance and microbiome disruption |
| Cagetti MG; Cocco F; Wierichs RJ; Wolf TG; Salerno C; Arghittu A; Campus G | 2022 | Italy, Switzerland, Germany, Russian Federation | Randomised controlled trial (triple-blind, 2-year, cluster RCT) | Primary research (clinical trial) | School-based setting (Italy; cluster randomisation by school class) | Children aged 4-5 and 6-7 years | 610 enrolled (518 completed) | Fluoride-substituted hydroxyapatite (HAF) + fluoride (1000/1450 ppm) in chitosan matrix | Toothpaste (supervised and home use; 3x daily brushing) | Sodium monofluorophosphate fluoride toothpaste (1000/1450 ppm) | Caries incidence and progression (ICDAS); risk ratio (RR); number needed to treat (NNT); survival analysis of teeth | HAF toothpastes significantly reduced caries increment compared to conventional fluoride toothpastes over 24 months; ~38-39% RR reduction in primary dentition and ~29% in permanent dentition; greater effect on both new lesion prevention and progression control | Cluster design and reliance on home compliance; COVID-19 disruption affecting follow-up and supervision; limited control over at-home brushing behaviour; potential adherence variability |
| O'Hagan-Wong K; Enax J; Meyer F; Ganss B | 2022 | Canada / Germany | Narrative review | Review article | Mixed (in vitro, in situ, in vivo, and clinical studies) | Human, laboratory, and clinical populations across included studies (children, orthodontic patients, general populations) | Not applicable | Hydroxyapatite (HAP; micro- and nanocrystalline forms) | Hydroxyapatite-containing toothpastes (various formulations; fluoride-free or combined systems) | Comparators varied (fluoride toothpastes, placebo, other remineralising agents) | Enamel remineralisation; caries prevention; surface hardness; lesion progression (ICDAS/DMFT); biofilm effects; dentinal hypersensitivity | HAP toothpastes demonstrate remineralisation potential comparable or equivalent to fluoride in many in vitro, in situ, and limited clinical studies; evidence suggests non-inferiority for caries prevention and potential advantages (deeper lesion penetration, biomimetic repair, no fluorosis risk) | Limited number of high-quality RCTs; heterogeneity in study designs and formulations; reliance on in vitro/in situ data; unclear long-term clinical effectiveness and impact on dentin lesions |
| Sari YW; Nuzulia NA; Wahyuni WT; Bahtiar A; Saputra A; Subroto MHA; Ariesanti Y; Syafitri U; Bachtiar I | 2022 | Indonesia | In vitro experimental laboratory study (formulation optimisation + remineralisation + antimicrobial testing) | Primary laboratory study | In vitro (extracted human teeth + S. mutans culture assays) | Extracted human premolars (tooth samples) + Streptococcus mutans (microbial model) | n=20 teeth (80 specimens); 20 toothpaste formulations tested | Nanohydroxyapatite (nanoHAP) + Curcuma aeruginosa extract (essential oil) | Experimental toothpaste formulations (20 variants; optimisation via mixture design; application to demineralised enamel; antimicrobial assays) | Chlorhexidine gluconate (0.2%); commercial toothpastes (standard, HAP, antibacterial, antibiofilm, herbal) | Remineralisation (SEM morphology; grey level mineral density change); antibacterial activity (zone of inhibition, MIC, MBC); antibiofilm activity (IC50 biofilm degradation) | Optimised formulation (OF1) induced enamel remineralisation (increased mineral density on SEM) and showed antibacterial activity comparable to chlorhexidine; antibiofilm activity superior to control (lower IC50); ingredient interactions influenced efficacy | In vitro design; complex multi-component formulation limits attribution of effect; no clinical validation; variability across formulations; limited direct comparison with standard fluoride dentifrices; short-term outcomes only |
| Gupta A; Gallagher JE; Chestnutt IG; Godson J | 2021 | UK | Cross-sectional survey study (market analysis of dentifrices) | Observational study (cross-sectional) | Retail market analysis (supermarkets, pharmacies, online retailers) | Dentifrice products (consumer products; no human participants) | n=500 products across 95 brands | Not applicable (product survey; multiple active ingredients including fluoride, nHAp, xylitol, herbal agents) | Various dentifrice formulations (toothpastes, gels, powders, tablets) | Not applicable | Fluoride concentration (ppm); product characteristics (labelling, flavour, marketing terms) | 31% of dentifrices contained no fluoride; 60% contained ≥1000 ppm fluoride; 45% ≥1350 ppm; increasing diversity of fluoride-free “natural/organic” products; wide variation in formulations and marketing claims | Descriptive cross-sectional design; no clinical outcomes; reliance on product labelling accuracy; UK-specific market (limited generalisability); no assessment of efficacy or bioavailability |
| Farhad F; Kazemi S; Bijani A; Pasdar N | 2021 | Iran | In vitro experimental study (pH cycling remineralisation model) | Primary laboratory study | In vitro (extracted human enamel; simulated oral environment) | Extracted human premolars (non-carious; orthodontic extractions) | n=90 teeth (sectioned into buccal/lingual segments; n=15 per group) | Theobromine (200 mg/L; 1.1 mol/L reported) | Solution immersion (1 min pre/post pH cycling; 7-day remineralisation-demineralisation cycle) | 0.05% sodium fluoride solution; artificial saliva control | Surface microhardness (Vickers hardness); calcium content (EDS analysis) | All groups showed remineralisation; theobromine produced significantly greater increase in surface microhardness (36.56±4.95 vs 23.25±3.92 for NaF) and highest calcium deposition (3.82±1.83 wt%); superior to fluoride under study conditions | In vitro design (limited clinical translation); short duration (7 days); single-agent comparison (no toothpaste formulation); variability in protocols vs other studies; conflicting evidence in literature; requires in vivo validation |
| Nazemi Salman B; Sallah S; Abdi F; Salahi S; Rostamizadeh K; Basir Shabestari S | 2021 | Iran | In vitro experimental study (well diffusion, MIC, MBC assays) | Primary laboratory study | In vitro (bacterial culture assays) | Streptococcus mutans, Streptococcus sobrinus, Streptococcus salivarius, Lactobacillus acidophilus, Enterococcus faecalis | Not explicitly stated (standardised microbial cultures; McFarland 0.5) | Nigella sativa nanoemulsion (various concentrations) | Nanoemulsion antimicrobial testing (disk diffusion, broth dilution) | 0.2% chlorhexidine mouthwash; sterile serum control | Growth inhibition zone (mm); minimum inhibitory concentration (MIC); minimum bactericidal concentration (MBC) | Nigella sativa nanoemulsion significantly inhibited growth of all tested cariogenic bacteria, but with smaller inhibition zones than chlorhexidine; MIC/MBC values higher (less potent) than chlorhexidine; Enterococcus faecalis most resistant, Lactobacillus acidophilus most susceptible | In vitro design; lack of clinical validation; variability in nanoemulsion formulation and concentrations; limited standardisation; results may not translate to in vivo oral biofilm complexity |
| Badekova KZ; Atazhanova GA; Kacergius T; Akhmetova SB; Smagulov MK | 2021 | Kazakhstan and Lithuania | In vitro preclinical experimental study (formulation + antimicrobial testing) | Primary laboratory study | In vitro (biofilm assay + disk diffusion antimicrobial testing) | Streptococcus mutans (biofilm model); Staphylococcus aureus, Bacillus subtilis, Escherichia coli, Pseudomonas aeruginosa, Candida albicans | Not clearly specified (biofilm assays + standard microbial strains) | Origanum vulgare essential oil (rich in carvacrol) + ethanol extract | Dental gel formulations (5 variants: ACDG1-ACDG5; sodium CMC-based gel) | Benzylpenicillin (bacteria); nystatin (fungi); untreated controls | S. mutans biofilm biomass (colorimetric assay); inhibition zone diameter (mm); antimicrobial activity grading | O. vulgare essential oil significantly inhibited S. mutans biofilm (≈98% reduction at ≥4 mg/ml); formulated gels (especially ACDG1 and ACDG3) showed high antimicrobial activity against gram-positive bacteria and Candida, moderate activity against gram-negative bacteria; ACDG3 identified as optimal anti-caries formulation | In vitro design; lack of clinical validation; variability between gel formulations; limited standardisation of sample size; biofilm assessment method limitations (destructive, limited structural insight) |
| Jacob B; Malli Sureshbabu N; Ranjan M; Ranganath A; Siddique R | 2021 | India | Randomised controlled trial (triple-blind, parallel-group RCT; 4-week follow-up) | Primary research (clinical trial) | Clinical setting (dental hospital; Chennai, India) | High caries risk adults (19-59 years; DMFT >6) | n=60 (30 PPE mouthwash; 30 chlorhexidine control) | Pomegranate peel extract (PPE) oral rinse | Mouthwash (10 ml nightly for 4 weeks) | Chlorhexidine mouthwash (0.2%) | Salivary bacterial load (qPCR): Streptococcus mutans, Lactobacilli, Veillonella (copies/µl at baseline, 2 and 4 weeks) | PPE disrupted all tested microorganisms but was less effective than chlorhexidine for S. mutans reduction; no significant differences overall between groups; CHX showed significant reduction in S. mutans over 4 weeks, while PPE showed variable but moderate antimicrobial effects | Short follow-up (4 weeks); adult population only (limited paediatric applicability); variability in microbial response; lack of long-term outcomes; ecological effects on oral microbiome not fully explored |
| Kasemkhun P; Rirattanapong P | 2021 | Thailand | In vitro experimental study | Primary laboratory study | In vitro (pH-cycling model on artificial enamel caries) | Extracted primary incisors (sound teeth; paediatric enamel model) | n=50 teeth (10 per group) | CPP-ACP; nanohydroxyapatite (nHAp); calcium glycerophosphate + calcium lactate (CaGP + CL) | Toothpaste slurries (1:3 dilution; twice daily application during 7-day pH-cycling) | 1000 ppm fluoridated toothpaste; deionised water control | Surface microhardness (Vickers hardness number, VHN); % surface microhardness recovery (%SMHR) | CPP-ACP and nHAp toothpastes showed remineralisation comparable to 1000 ppm fluoride (no significant difference); CaGP + CL toothpaste significantly less effective; all test groups superior to control | In vitro model (limited clinical translation); short duration (7-day pH cycling); absence of saliva/biofilm dynamics; unknown concentration of some active ingredients (commercial formulations) |
| Arjun DS; Bhat SS; Hegde SK; Bhat VS; Rao HTA; Ramdas SS | 2021 | India | In vitro experimental study | Primary laboratory study | In vitro (artificial enamel caries model; DIAGNOdent assessment) | Extracted premolars (non-carious teeth; enamel specimens) | n=51 teeth (17 per group) | CPP-ACP; tricalcium phosphate (customised dentifrice) | Topical application (3 min, twice daily; 30-day remineralisation protocol in artificial saliva) | Artificial saliva control | DIAGNOdent fluorescence readings (baseline, post-demineralisation, 15 days, 30 days) | Both CPP-ACP and tricalcium phosphate dentifrice demonstrated significant remineralisation vs control; no significant difference between agents; CPP-ACP showed greater magnitude of remineralisation and considered gold standard | In vitro design; lack of oral biofilm/saliva dynamics; short-term outcomes (30 days); customised dentifrice formulation limits reproducibility; no comparison with fluoride dentifrice |
| Amaechi BT; Phillips TS; Evans V; Ugwokaegbe CP; Luong MN; Okoye LO; Meyer F; Enax J | 2021 | USA / Nigeria / Germany | In vitro experimental study (pH-cycling caries model) | Primary laboratory study | In vitro (root dentin blocks; pH-cycling model simulating demineralisation/remineralisation) | Human extracted teeth (root dentin blocks) | n=60 blocks (20 per group) | Hydroxyapatite (10% HAP toothpaste) | Toothpaste slurry (1:3 dilution; twice-daily application within 7-day pH-cycling regimen) | 1450 ppm fluoride toothpaste (NaF); artificial saliva control | Mineral loss (ΔZ, transverse microradiography); % inhibition of demineralisation | HAP and fluoride toothpastes showed no significant difference in preventing root demineralisation; HAP demonstrated numerically greater inhibition (21%) vs fluoride (6%); both superior to control; HAP considered effective alternative for root caries prevention | In vitro design; absence of oral biofilm and salivary dynamics; aggressive demineralisation model; short duration (7 days); requires clinical validation |
| Durhan MA; Ozsalih S; Gokkaya B; Kulan PY; Kargul B | 2021 | Turkey | Randomised clinical trial (preliminary study) | Primary clinical study | Clinical (paediatric population; 1-month intervention) | Children aged 4-5 years with early childhood caries (ECC) | n=26 children (13 per group; 260 teeth total) | Theobromine (Theodent Kids toothpaste) | Toothbrushing twice daily for 1 month | 500 ppm fluoride toothpaste (Colgate Kids) | Laser fluorescence (DIAGNOdent) for remineralisation; salivary pH; buffering capacity; Streptococcus mutans levels | Both theobromine and fluoride toothpastes significantly improved enamel remineralisation with no significant difference between groups; theobromine additionally increased salivary pH and buffering capacity and reduced S. mutans levels | Small sample size; short follow-up (1 month); preliminary study; low fluoride comparator (500 ppm); limited power to detect between-group differences |
| Mazur M; Ndokaj A; Jedlinski M; Ardan R; Bietolini S; Ottolenghi L | 2021 | Italy; Poland | Systematic review and meta-analysis | Systematic review (RCT synthesis) | Mixed (clinical trials; periodontal + caries outcomes) | Humans (children and adults across included RCTs) | n=870 participants (18 RCTs included) | Green tea (Camellia sinensis; catechins e.g. EGCG) | Various (mouth rinse, gel, toothpaste, chewing gum, strips, capsules, drinking tea) | Placebo, chlorhexidine, sodium fluoride, triclosan, or no treatment | Gingival Index (GI); Plaque Index (PI); Bleeding indices (GBI, BOP); Probing Pocket Depth (PPD); Clinical Attachment Loss (CAL); bacterial counts (Streptococcus mutans, Lactobacillus spp.) | Green tea showed medium positive effects on gingival inflammation (GI, PI, GBI, BOP) and large effect for reducing PPD; small reductions in S. mutans and Lactobacillus; however, overall evidence insufficient to recommend as first-line treatment, especially for caries | High heterogeneity across studies; variable formulations and delivery methods; inconsistent outcomes; risk of bias in included trials; lack of standardised dosing (e.g. EGCG); limited caries-specific outcomes |
| Limeback H; Enax J; Meyer F | 2021 | Canada / Germany | Systematic review and meta-analysis | Systematic review (RCT synthesis) | Mixed (clinical trials; caries-focused + proxy outcomes) | Humans (children and adults across included studies) | n=291 studies identified; 22 included; 5 clinical caries trials (4 RCTs; 3 in meta-analysis) | Biomimetic hydroxyapatite (HAP) | Toothpaste, mouthwash, gel (fluoride-free formulations) | Fluoride-containing products, placebo, or no treatment | Caries incidence (e.g. ICDAS, DMFT); remineralisation outcomes; plaque/biofilm measures; bacterial counts | HAP provided ~17% reduction in caries risk in meta-analysis of 3 RCTs; multiple trials showed non-inferiority to fluoride; overall evidence supports HAP as an effective fluoride-free anticaries agent, particularly for children | Small number of comparable RCTs; heterogeneity in study design, formulations, and outcomes; limited ability to assess heterogeneity statistically; inclusion of proxy outcomes alongside true caries outcomes; potential conflicts of interest (industry involvement) |
| Amaechi BT; Alshareif DO; Azees PAA; Shehata MA; Lima PP; Abdollahi A; Kalkhorani PS; Evans V; Bagheri A; Okoye LO | 2021 | USA and Nigeria | Randomized, double-blind, crossover in situ study | Primary research (in situ experimental study) | Clinical research setting (intra-oral appliance model) | Adults (18-50 years; high caries risk) | n=32 enrolled; n=30 completed | Nano-hydroxyapatite (5% nanoHAP) | Toothpaste (5% nanoHAP) + adjunct dental lotion (5% nanoHAP, post-brushing rinse) | Placebo dental lotion (0% nanoHAP) used after nanoHAP toothpaste | % remineralisation (TMR); mineral loss (ΔZ); demineralisation inhibition in sound enamel | Mineral gain significantly higher with nanoHAP lotion vs placebo (58.4% vs 37.7%, p<0.001); both groups showed remineralisation due to nanoHAP toothpaste; no demineralisation observed in sound enamel in either group | In situ model limits generalisability to real-world clinical outcomes; no fluoride comparator; no placebo toothpaste arm (all participants used nanoHAP toothpaste); short duration; potential industry funding bias (Sangi Co., Ltd.) |
| Wiatrak K; Morawiec T; Roj R; Kownacki P; Nitecka-Buchta A; Niedzielski D; Wychowanski P; Machorowska-Pieniazek A; Cholewka A; Baldi D; Mertas A | 2021 | Poland | Randomized controlled clinical study (parallel group) | Primary research (clinical trial) | Clinical setting (prosthodontic patients with removable dentures) | Adults (41-82 years; removable partial denture wearers) | n=50 (25 intervention, 25 control) | Tea tree oil (TTO, 1.0%) + ethanolic extract of propolis (EEP, 1.0%) | Toothpaste containing TTO + EEP (plus base ingredients) | Placebo toothpaste (identical base, no active antimicrobial ingredients) | Oral hygiene indices (API, OHI-s, DPI); gingival inflammation (mSBI); oral microbiota (bacterial/fungal strains) | Significant improvement in oral hygiene indices (API, OHI-s) and gingival health (mSBI reduced to 0% bleeding in study group at 28 days); reduction in total microbial strains (108→80 vs 100→104 control); decreased Candida albicans counts in intervention group | Short duration (28 days); surrogate outcomes (indices/microbiology, not caries); adult denture population (limited generalisability to children); multi-component intervention (cannot isolate active ingredient effect); no fluoride comparator; potential Hawthorne effect from hygiene instruction |
| Rajendran R; Nair KR; Sandhya R; Krishnan AV; Anilkumar A; Rakhi PV | 2021 | India | Experimental in vitro comparative study (3 groups; demineralisation + remineralisation cycles) | Primary laboratory study | In vitro | Extracted human premolars (enamel specimens) | n=90 specimens (4×4×1 mm); 3 groups n=30 each | Strontium‑doped nano‑hydroxyapatite (lab‑synthesised Sr‑nHAp); CPP‑ACP (commercial); regular dentifrice (control) | Sr‑nHAp paste (powder + sodium alginate, sorbitol, SLS, glycerine, water); CPP‑ACP topical cream/toothpaste; motorized brushing 3 min twice daily ×28 days; stored in artificial saliva | Regular toothpaste (control) | SEM surface morphology; EDX elemental analysis (mean Ca; mean P; Ca/P); ANOVA + Tukey post hoc | Both CPP‑ACP and Sr‑nHAp produced significant remineralisation vs control; Sr‑nHAp > CPP‑ACP > control in post‑treatment Ca and P; Sr‑nHAp showed smoother SEM surface | In vitro model; McInnes chemical demineralisation; short duration (28 days); enamel specimens only; no in vivo/clinical or toxicology data; limited generalisability |
| Paszynska E; Pawinska M; Gawriolek M; Kaminska I; Otulakowska-Skrzynska J; Marczuk-Kolada G; Rzatowski S; Sokolowska K; Olszewska A; Schlagenhauf U; May TW; Amaechi BT; Luczaj-Cepowicz E | 2021 | Poland, Germany, USA | Multicentre double‑blind randomized active‑controlled parallel‑group trial | Primary study (randomised clinical trial) | Clinical (multicentre pediatric clinics) | Children aged 3-7 yrs with full primary molars and ≥1 restored primary molar | ITT n=207; PP n=177 | 10% microcrystalline hydroxyapatite (HAP); amine fluoride 500 ppm (control) | Toothpaste (HAP test toothpaste identical to commercial Kinder Karex; control amine‑fluoride toothpaste); brushing 3×/day with provided electric (am/pm) and manual (noon) brushes for 336 days | 500 ppm amine fluoride (amine fluoride toothpaste) | Primary: proportion with new/progressed enamel caries ICDAS ≥1 (per tooth/surface); Secondary: ICDAS ≥2; Plaque Control Record (PCR); Modified Gingival Index (GI); adverse events | HAP was non‑inferior to 500 ppm amine‑fluoride for preventing new/progressed enamel caries (ICDAS ≥1 and ≥2) in PP and ITT analyses; PCR and GI improved similarly; no serious/severe AEs observed, supporting HAP as an effective biomimetic alternative in children. | Limitations: manufacturer funding and no dietary control; supervised 3×/day brushing with electric brushes may reduce generalisability to typical home use; non‑inferiority design prevents claims of superiority and population had high baseline caries risk. |
| Chhaliyil P; Fischer KF; Schoel B; Chhalliyil P | 2020 | India | Randomized, controlled intervention (3‑arm school‑based trial) | Primary study (randomized trial) | School clinic (Andavar Porayar School, Tamil Nadu) | Healthy children aged 10-12 yrs | 45 (15 per group) | None for GIFTS (water); sodium‑fluoride (~100 mg) for BT; activated nano‑charcoal (~100 mg) for CT | GIFTS: gum/tooth rubbing with index finger + tongue cleaning + water swishing after each meal/snack/drink (~6-8×/day); BT: brushing + tongue cleaning twice daily with NaF toothpaste; CT: GIFTS twice daily with charcoal, plus tongue cleaning | BT (brushing + tongue cleaning twice daily with NaF toothpaste) | Total bacterial load by qPCR (saliva, plaque, tongue); 16S rRNA V3-V4 metagenomics (QIIME/Greengenes); qPCR for S. mutans and A. actinomycetemcomitans | GIFTS produced larger reductions in total salivary bacterial load than BT (P<0.004 vs P<0.02) and reduced early colonizers (e.g., Streptococcus) in plaque versus BT/CT; all methods reduced Fusobacterium and some red‑complex taxa; frequent biofilm disruption (GIFTS) was more effective than twice‑daily brushing at limiting early colonizers over 10 days. | Small sample (n=45) and short duration (10 days); no clinical endpoints (no caries/gingivitis outcomes); unequal cleaning frequency between arms (frequency vs method confounding); single school site limits generalisability. |
| Amaechi BT; AbdulAzees PA; Okoye LO; Meyer F; Enax J | 2020 | USA/Nigeria/Germany | Laboratory pH‑cycling in vitro study | Primary study (in vitro) | Laboratory (bovine tooth block model) | Bovine enamel blocks (from 20 bovine teeth) | 60 blocks (20 per group) | 15% hydroxyapatite (HAP) gel; 12,500 ppm F− (amine fluoride) gel; artificial saliva (control) | Gels applied as thin ribbon to enamel: HAP gel 3 min once daily; fluoride gel 3 min once weekly; otherwise stored in artificial saliva during 28‑day pH‑cycling | Fluoride gel (12,500 ppm amine fluoride) and artificial saliva | % mineral gain (remineralization) by transverse microradiography (TMR); pre/post microradiographic lesion images and lesion depth/ΔZ | 15% HAP gel produced mean ~39% remineralization (±7%) and high‑F gel ~41% (±11%); both significantly greater than artificial saliva (~6%); no significant difference between HAP and high‑F (non‑inferior); microradiographs showed HAP produced more homogeneous/deeper remineralization while fluoride produced predominantly surface‑zone remineralization. | Limited by in vitro pH‑cycling model lacking biofilm/biological factors and using bovine (not human) enamel; aggressive demineralization regimen and differing application schedules (daily HAP vs weekly fluoride) may limit clinical extrapolation; company‑coded products (sponsor involvement) and need for clinical trials. |
| Selvaraj K; Bharath N; Natarajan R; Dinesh S; Murugesan S; Selvaraj S | 2020 | India | Randomised controlled clinical study (parallel-group, 60-day follow-up) | Primary research (clinical trial) | Clinical setting (dental college population; in vivo saliva analysis) | Adults aged 18-30 years (moderate-high caries risk population) | n=60 (30 probiotic toothpaste; 30 neem toothpaste) | Probiotic toothpaste (Lactobacillus-based; PerioBiotic) and neem-based toothpaste (Azadirachta indica) | Toothpaste use twice daily for 60 days | Active comparator (probiotic vs neem toothpaste; no fluoride/placebo control) | Salivary Streptococcus mutans counts (CFU; measured at baseline, 15, 30, 60 days) | Both probiotic and neem toothpastes significantly reduced S. mutans counts over 60 days; gradual intragroup reduction observed; no statistically significant difference between groups; neem showed strong antimicrobial activity attributed to phytochemicals | Short duration (60 days); surrogate outcome (bacterial counts, not caries); no true control (no placebo/fluoride comparator); limited generalisability (young adult population); compliance-dependent; unclear blinding/randomisation details |
| Nordin A; Bin Saim A; Ramli R; Abdul Hamid A; Mohd Nasri NW; Bt Hj Idrus R | 2020 | Malaysia | Systematic review | Narrative/systematic review (mixed evidence synthesis) | Mixed (in vitro, in vivo, and clinical studies across multiple settings) | Humans, animal models, and laboratory specimens across included studies | Not applicable (review of studies published 2010-May 2020) | Salvadora persica (miswak; multiple active compounds including flavonoids, alkaloids, tannins, benzyl derivatives) | Multiple delivery forms (chewing stick, toothpaste, mouthwash, gel, varnish, chewing gum, extracts) | Various (fluoride toothpaste, chlorhexidine, placebo, no treatment, other herbal agents) | Plaque index (PI); gingival index (GI); bleeding indices; caries outcomes (DMFT); microbial counts; remineralisation measures; wound healing outcomes | Miswak demonstrated consistent positive effects across studies including anti-plaque, anti-gingivitis, antimicrobial, anticariogenic, remineralisation potential, and wound healing benefits; effectiveness comparable or sometimes superior to conventional agents depending on formulation and context | Heterogeneity of included studies (designs, populations, formulations); lack of standardisation of miswak delivery/dose; reliance on surrogate outcomes in many studies; limited high-quality long-term RCT evidence for caries endpoints |
| Tharakan AP; Pawar M; Kale S | 2020 | India | Systematic review | Systematic review (clinical study synthesis in paediatric population) | Mixed (clinical trials and RCTs in children) | Children aged 3-15 years across included studies | n=6 studies (10 estimates; small sample sizes across studies, range ~6-66 participants) | Licorice (Glycyrrhiza glabra; root extract) | Various (lollipops, mouthwash, dentifrice, gels) | Comparators varied (placebo, no treatment, other interventions; some single-arm designs) | Streptococcus mutans counts; caries prevention indicators (indirect); salivary pH changes | Licorice demonstrated antimicrobial activity with reduction in S. mutans counts and modulation of oral biofilm/pH; lollipop delivery particularly effective and well accepted in children; potential role in caries prevention through ecological mechanisms | Limited number of included studies; small sample sizes; short intervention durations; heterogeneity in study design and delivery methods; reliance on surrogate outcomes (S. mutans rather than caries incidence); limited long-term clinical evidence |
| de Oliveira Carvalho I; Purgato GA; Piccolo MS; Pizziolo VR; Coelho RR; Diaz-Munoz G; Alves Nogueira Diaz M | 2020 | Brazil | In vitro experimental study | Primary laboratory study | In vitro (biofilm + antimicrobial assays; dentifrice formulation testing) | Bacterial strains: Streptococcus mutans, Staphylococcus aureus, Enterococcus faecalis, Lactobacillus lactis | Not explicitly stated (standardised microbial assays; triplicate testing) | Essential oils (cinnamon, clove, oregano, thyme; active compounds e.g. cinnamaldehyde, eugenol, thymol, carvacrol) | Toothpaste formulations (3% and 5% essential oils; alone and in combination) | 0.12% chlorhexidine gluconate (positive control); base toothpaste without essential oils | Minimum inhibitory concentration (MIC); inhibition zone (mm); % biofilm disruption; antibacterial activity (hole-plate assay) | Cinnamon, clove, oregano, and thyme essential oils demonstrated strong antibacterial and antibiofilm activity; several toothpaste formulations completely disrupted S. mutans biofilms and showed effects comparable to chlorhexidine; thyme showed synergistic interaction with chlorhexidine | In vitro design; no clinical validation; variability in formulations and concentrations; absence of fluoride comparator; surrogate microbiological outcomes only; limited translation to real-world oral environment |
| Chandhru TP; Anusha VR; Peedikayil FC; Gufran Ahmed MB; Kottayi S; Narasimhan D | 2020 | India | Ex vivo experimental study | Primary laboratory study (clinical isolate-based) | Laboratory setting (Candida albicans cultured from ECC patients) | Children aged 3-6 years with early childhood caries (ECC) | n=60 (10 per toothpaste group; clinical isolates analysed) | Commercial children’s toothpastes (various active ingredients including sodium fluoride, neem, meswak, coconut oil, triclosan) | Toothpaste slurries (1:1 dilution primarily; agar well diffusion method) | Distilled water (negative control) | Zone of inhibition (mm) against Candida albicans | All toothpastes demonstrated antifungal activity; Pediflor Kids toothpaste showed highest inhibition (18.20±2.13 mm) and Aloe Dent lowest (8.96±0.86 mm); antifungal effects attributed to active ingredients such as fluoride, neem, meswak, coconut oil, and triclosan | Ex vivo/in vitro design; surrogate outcome (Candida inhibition, not caries); mixed formulations (multi-ingredient, cannot isolate effects); lack of fluoride-free comparator standardisation; limited clinical translation; dilution and diffusion variability may affect results |
| Sebelemetja M; Moeno S; Patel M | 2020 | South Africa | In vitro experimental study (nanoparticle-based) | Primary laboratory study (mechanistic + formulation science) | Laboratory setting (S. mutans cultures; nanoparticle drug-delivery model) | Bacterial strains (S. mutans reference + clinical isolates) | Not explicitly stated (triplicate assays across strains) | Dodonaea viscosa flavone (F5.1) stabilised PLGA-PEG nanoparticles | Nanoparticle formulation (flavone-loaded polymeric nanoparticles; controlled release system) | Chlorhexidine (positive control); DMSO/water (controls); blank nanoparticles | Minimum inhibitory concentration (MIC); acid production (pH); biofilm formation (% reduction); drug release profile; cytotoxicity (IC50) | F5.1 nanoparticles showed anti-S. mutans activity (MIC 1.56 mg/ml); significantly reduced acid production without reducing bacterial counts (anti-acidogenic effect); reduced biofilm formation by ~91-92%; demonstrated sustained release up to 12 hours with pH-dependent release profile | In vitro study; highly experimental formulation (nanoparticles not clinically available); surrogate outcomes (biofilm/acid, not caries); limited translational applicability; cytotoxicity concerns at higher concentrations; complex delivery system limits generalisability |
| Wang X(1), Li S(1), Li Y(1), Yu H(1), Qi W(1)(2), Wang J(1)(2). | 2025 | China | In vitro experimental study (dual-species biofilm model) | Primary laboratory study | In vitro (saliva-coated enamel-dentin discs; simulated brushing exposure) | Dual-species biofilm: Streptococcus mutans + Porphyromonas gingivalis | n=48 discs per group | Nano-hydroxyapatite (nHAp) | nHAp-containing toothpaste (twice daily exposure; simulated brushing regimen) | Fluoride toothpaste; domiphen bromide toothpaste; water control | pH (acidogenicity); bacterial counts (CFU); SEM morphology; CLSM biofilm viability | nHAp toothpaste showed the smallest pH drop (best resistance to acidogenicity) and significantly reduced bacterial counts compared with control; SEM and CLSM confirmed reduced biofilm density and viability; overall strongest antibacterial effect among tested toothpastes | In vitro model; simplified dual-species biofilm (does not reflect full oral microbiome); no caries or clinical endpoints; short duration (96 h); toothpaste exposure conditions may not fully replicate real-world brushing dynamics |
| Rafeie N(#)(1), Salimi Y(#)(2), Aghamir ZS(3), Amini A(4), Taheri H(5), Sadreddini S(6), Kamali F(3), Akbarian G(7), Azizi N(3), Bagherianlemraski M(8), Valizadeh M(9), Alimohammadi F(10), Sedighnia N(11), Qadirifard M(#)(12), Naziri M(#)(13). | 2025 | United States, Iran, Russia, Canada, India, Brazil, Egypt | Systematic review | Systematic review of RCTs (clinical evidence synthesis) | Clinical (in vivo; mouthwash interventions in human participants) | Children and adults (approx. 5-50 years; many studies in paediatric populations) | n=7 studies (all RCTs; sample sizes typically 20-84 participants per study) | Punica granatum (pomegranate; polyphenols incl. punicalagin, ellagic acid, tannins) | Mouthwash (varied formulations: aqueous, hydroalcoholic; peel, juice, or whole-fruit extracts) | 0.2% chlorhexidine (primary comparator); other herbal rinses; saline; distilled water | Streptococcus mutans counts; plaque index; salivary pH; enzymatic markers (alpha-glucosidase, ceruloplasmin) | Pomegranate mouthwash consistently reduced S. mutans counts and plaque indices; effects often comparable but generally slightly inferior to chlorhexidine, though some studies showed equivalence or superiority; also increased salivary pH and influenced enzymatic pathways linked to cariogenesis; no adverse effects reported | Limited number of studies; heterogeneity in formulations, concentrations, and protocols; short follow-up durations; reliance on surrogate outcomes; inconsistent reporting of adverse events; unclear which plant component drives effect |
| Michałowski K(1), Górski B(2), Brodzikowska A(1). | 2025 | Poland | Randomised controlled trial (double-blind) | Primary clinical trial (in vivo human study) | Outpatient dental clinic (clinical setting) | Adults with gingivitis (18-65 years; ≥20 teeth; non-smokers) | n=113 enrolled; n=104 analysed (approx. 52 per group) | Sinapis alba (white mustard extract; glucosinolates → isothiocyanates) | Toothpaste (0.5% mustard extract; fluoride-free formulation; twice daily brushing for 4 weeks) | Placebo toothpaste (identical fluoride-free base without mustard extract) | Plaque index (PI); approximal plaque index (API); gingival index (GI); bleeding on probing (BoP); salivary Streptococcus mutans and Lactobacillus spp. (≥/≤10⁵ CFU/mL) | Significant reductions in all clinical indices in both groups, but greater improvements in experimental group (e.g., PI reduction greater, p=0.041; BoP reduction greater, p=0.037); marked reduction in S. mutans (51.9%→19.2%) and Lactobacillus spp. (55.8%→9.6%) above threshold vs smaller reductions in control; statistically significant association between mustard toothpaste use and bacterial reduction | Short duration (4 weeks); no comparison with fluoride-containing toothpaste; adult population (limited paediatric applicability); surrogate outcomes (bacterial load vs caries incidence); potential allergenicity of mustard; modest sample size |
| Jungbauer G, Lechner R, Stähli A, Sculean A, Eick S | 2025 | Switzerland / Germany | In vitro experimental study (multi-species biofilm model) | Laboratory study (in vitro) | Controlled laboratory setting (multi-species supragingival biofilm model) | Defined oral bacterial strains (including Streptococcus mutans, S. sobrinus, Fusobacterium nucleatum, Porphyromonas gingivalis, etc.); gingival fibroblasts for cytotoxicity assays | Not applicable (replicated in vitro experiments; ≥12 replicates per condition) | Manuka honey (methylglyoxal-mediated activity) + propolis (flavonoids, phenolics) ± fluoride | Toothpaste formulations (Manuka honey 4-5% with either 0.2% propolis or fluoride; tested at 5-20% dilutions; applied to established biofilm for 1 min or surface coating for biofilm formation) | Toothpaste base without active ingredients (TP con); commercial toothpaste (Colgate Total); distilled water (negative control) | Minimum inhibitory concentration (MIC); colony-forming units (CFU); metabolic activity (resazurin assay); biofilm mass (crystal violet); biofilm formation inhibition; gingival fibroblast viability (MTT assay) | All formulations showed antibacterial activity (MIC ≤0.04%); significant reduction in CFU and metabolic activity in preformed biofilm; Manuka + propolis showed greater CFU reduction than base; concentration-dependent inhibition of de novo biofilm formation; commercial toothpaste most effective overall; biofilm mass largely unchanged; cytotoxicity increased with concentration but similar across formulations | In vitro design (no clinical applicability); no mechanical brushing component; biofilm mass not reduced despite bacterial reduction; base formulation also active (confounding effect of excipients); high concentrations required for effect; cytotoxicity observed at higher concentrations; short exposure models |
| Wang Y(1)(2), Chen S(3), Zhang M(4), Chen L(1)(2)(5), Zhou C(6)(7), Tan S(8)(9). | 2024 | China | In vitro experimental materials science study (dentin model + cell culture) | Laboratory study (in vitro + ex vivo dentin + cell assays) | Controlled laboratory setting (artificial saliva + citric acid cycling models; dentin slices; cell culture) | Human dentin samples (extracted teeth, n=49 dentin sheets); human dental pulp stem cells (DPSCs) | Not applicable (laboratory replicates; n≈3-10 per condition depending on assay) | Nano hydroxyapatite-silica (nHASi; core-shell: nHA core + silica shell) | Toothpaste formulation containing nHASi; hydrosol and powder forms tested; cyclic de/remineralisation conditions (citric acid pH ~2.2 + artificial saliva) | Nano hydroxyapatite (nHA) alone; fluoride toothpaste; commercial desensitising toothpaste (Sensodyne); untreated controls | Ion release kinetics (Ca2+, PO43-, SiO32-); SEM/TEM imaging; XRD; biofilm mineralisation; acid resistance (citric acid exposure); dentin tubule occlusion rate; cytocompatibility (CCK-8, live/dead); odontogenic differentiation markers (DMP-1, DSPP) | nHASi demonstrated sustained Ca/P ion release and biomineralisation; superior acid resistance vs nHA (silica shell preserved structure under acidic challenge); enhanced dentin tubule occlusion over repeated de/remineralisation cycles (highest among all groups); deeper tubule penetration and HA crystal formation; favourable cytocompatibility; increased odontogenic differentiation vs nHA | In vitro design (no clinical validation); animal model validation not performed; no direct pain/hypersensitivity outcomes; brushing/mechanical forces not fully replicated; high-concentration laboratory conditions; limited translational evidence for long-term clinical effectiveness |
| Atazhanova GA(1), Levaya YK(1), Badekova KZ(1), Ishmuratova MY(2), Smagulo MK(2), Ospanova ZO(3), Smagulova EM(1). | 2024 | Kazakhstan | Narrative review of in vitro and limited in vivo studies on plant extracts, essential oils, and phytochemicals inhibiting Streptococcus mutans biofilm formation | Review (literature synthesis; microbiological, molecular, and biochemical evidence) | Not applicable (synthesis of laboratory and preclinical studies; occasional clinical references) | Not applicable (S. mutans models across included studies; biofilm, planktonic cultures, and gene expression systems) | Not applicable (review of multiple studies; no single sample size) | Multiple plant-derived compounds (e.g., polyphenols, flavonoids, tannins, essential oils, terpenes, catechins) | Various delivery contexts discussed (toothpastes, mouthwashes, gels, extracts, essential oil formulations) | Comparators across included studies include chlorhexidine (CHX), fluoride formulations, antibiotics, and untreated controls | Inhibition of biofilm formation; bacterial adhesion; EPS synthesis; glucosyltransferase activity; quorum sensing; gene expression (gtfB, gtfC, gtfD, etc.); acid production; bacterial viability; biofilm biomass (crystal violet); CLSM imaging; MIC/MBC assays | Plant-derived compounds inhibit S. mutans via multi-target mechanisms: reduced adhesion, suppression of EPS/glucan synthesis, disruption of quorum sensing, membrane damage, oxidative stress, and downregulation of virulence genes; essential oils and extracts demonstrated significant reductions in biofilm formation (often >90% in vitro) and, in some cases, comparable effects to CHX; potential applications in preventive oral care products highlighted | Predominantly in vitro evidence; heterogeneity in study design, concentrations, and formulations; lack of standardisation; limited clinical validation; potential toxicity/allergy concerns; unclear dosing and long-term effectiveness in real-world oral environments |
| Fernández CE(1), García-Manriquez NL(1), Zero D(2), Cury JA(3). | 2024 | Chile, USA, Brazil | Laboratory evaluation of fluoride (F) bioavailability and stability in commercially available activated charcoal-containing toothpastes | Experimental laboratory study (analytical chemistry; ion-selective electrode analysis) | Not applicable (product testing under laboratory conditions; fresh vs aged samples) | 20 commercial fluoridated charcoal-containing toothpastes (NaF and Na2FPO3 formulations), plus controls (NaF/silica, MFP/CaCO3, fluoride-free) | 20 toothpaste types (duplicates analysed; n=4 measurements per product) | Sodium fluoride (NaF) and sodium monofluorophosphate (Na2FPO3) within charcoal-containing formulations | Toothpaste (charcoal-containing dentifrices; silica-based abrasives) | Controls: conventional NaF/silica toothpaste, MFP/CaCO3 toothpaste, and fluoride-free toothpaste | Total fluoride (TF); total soluble fluoride (TSF = bioavailable F); ionic fluoride; monofluorophosphate fraction; % insoluble fluoride; pH; stability over time (fresh vs aged) | Most charcoal-containing toothpastes maintained bioavailable fluoride (TSF) close to total fluoride (~950-1,450 ppm), both fresh and after expiration; no significant reduction in fluoride stability over time; activated charcoal did not reduce fluoride bioavailability, likely due to neutral-basic pH (6.45-8.24) limiting adsorption; silica-based formulations preserved fluoride availability; only CaCO3/MFP control showed reduced TSF (~30% loss after aging) | Purely laboratory-based; no clinical outcomes (caries, remineralisation); variability in charcoal type/concentration unknown; does not assess abrasivity, wear, or long-term intraoral fluoride kinetics; generalisability limited to tested formulations |
| Jung K(1)(2), Kerzel P(1), Hara AT(3), Luka B(4), Schlueter N(4), Ganss C(1)(2). | 2024 | Germany, USA | In vitro laboratory experimental study . | In vitro experimental study (erosion/abrasion cycling model with human enamel) | Laboratory setting (cyclic erosion with citric acid ± brushing over 10 days) | Human enamel samples exposed to HAP toothpastes (with and without fluoride), HAP mouthrinse, SnF2 toothpaste, F/Sn mouthrinse, and negative control toothpaste | 256 enamel samples (n=16 per group across 16 groups) | Hydroxyapatite (HAP), HAP + fluoride (NaF), compared with stannous fluoride (SnF2) and fluoride/stannous mouthrinse | Toothpaste and mouthrinse (slurry exposure ± brushing) | Negative control (no active agent), SnF2 toothpaste, and F/Sn mouthrinse | Erosive tissue loss (µm); abrasion effects; free ionic fluoride; calcium concentration; REA/RDA; particle morphology (SEM/EDX) | HAP formulations (with or without fluoride) did not reduce erosive tissue loss compared to control; some increased tissue loss; brushing increased loss in all groups except F/Sn mouthrinse; F/Sn mouthrinse showed near-complete protection; HAP particles were amorphous, loosely bound, and easily removed; calcium in formulations reduced fluoride bioavailability in some cases | In vitro model limits clinical extrapolation; focuses on erosion rather than caries; short-term exposure; formulation variability (e.g., excipients like pyrophosphates) may confound effects; does not assess long-term remineralisation or patient outcomes |
| Shayegan A(1), Arab S(1), Makanz VM(1), Safavi N(1). | 2024 | Belgium | In vitro study | In vitro experimental study (QLF-based remineralisation model) | Laboratory setting (demineralisation-remineralisation cycling over 10 days) | Human enamel blocks from extracted permanent molars (sound teeth) | n=70 enamel samples (10 per group across 7 groups) | Calcium sodium phosphosilicate (CSP; NovaMin), turmeric (Curcuma), ginger (Zingiber officinale), fluoride (NaF) | Topical application (2× daily for 2 min over 10 days) | Fluoride toothpaste (1450 ppm NaF), fluoride varnish (22,600 ppm NaF), non-fluoride toothpaste, distilled water | Quantitative light-induced fluorescence (ΔF%); lesion depth and mineral loss before/after demineralisation and after remineralisation | Turmeric showed the greatest remineralisation (P<0.0001), comparable to fluoride varnish; CSP (NovaMin) also significantly improved remineralisation (P<0.01); fluoride toothpaste showed less effect than CSP; ginger showed no significant remineralisation effect | In vitro design; short-term (10 days); QLF surrogate outcome (not clinical caries); turmeric mechanism unclear; isolated compounds (e.g. ginger without synergistic agents like honey); variability in formulations; no biofilm/saliva simulation |
| Jila Torabi 1,2, Henrique Pedro Soares Luis 1,3, Gohar Mkrtchyan 1, Shohreh Derakhshan Alavijeh 1, Sameen Dezfoli 1, Michelle Hurlbutt 1. | 2024 | USA / Portugal | Single‑blind, randomized, parallel clinical trial | Single-blind randomised clinical trial (qPCR-based bacterial analysis) | Clinical setting (15-day intervention with salivary sampling pre/post) | Adults (dental hygiene and nursing students; ≥18 years) | n=30 enrolled; n=22 completed; n=10 analysed (5 per group with viable qPCR samples) | Cannabidiol (CBD; 300 mg nano-formulation) | Lozenge (slow-dissolving candy; once daily for 15 days) | Sugar-free candy (control) | Salivary Streptococcus mutans abundance (qPCR; relative gene expression vs total bacterial load) | Significant reduction in S. mutans levels in CBD group vs control (p=0.0299); post-intervention decrease observed only in experimental group; suggests antimicrobial effect of CBD on cariogenic bacteria | Small sample size (n=10 analysed); short duration (15 days); surrogate microbiological outcome (not caries); potential compliance issues; variability in saliva production; limited generalisability; potential broader microbiome disruption not assessed |
| Campus G(1), Cocco F(2), Wierichs RJ(3), Wolf TG(4), Salerno C(5), Arghittu A(2), Dettori M(2), Cagetti MG(6). | 2024 | Italy / Switzerland / Germany | Randomised, triple‑blind, parallel clinical trial | Triple-blind randomised controlled trial (cluster-randomised; 24-month follow-up) | Community/school-based clinical setting (supervised and home brushing) | Children aged 4-7 years | n=610 enrolled (cluster-randomised; ~14-16 per class) | Fluoride-substituted hydroxyapatite (HAF; 1000 ppmF and 1450 ppmF, with Mg/Sr/CO3 substitution in chitosan matrix) | Toothpaste (3× daily brushing for ≥2 min) | Conventional fluoride toothpaste (monofluorophosphate; 1000 ppmF and 1450 ppmF) | Plaque pH (acidogenicity curves; sucrose challenge); microbiological composition (checkerboard DNA-DNA hybridisation of cariogenic bacteria); caries (ICDAS) | HAF groups showed greater increase in minimum plaque pH (less acidogenic biofilm) and greater reduction in cariogenic bacteria (S. mutans, S. sobrinus, Lactobacillus spp.) compared to fluoride groups at 24 months (P≈0.02-0.04); both groups improved over time; overall evidence suggests modulation of biofilm towards less cariogenic profile but caries prevention effect remains inconclusive | Cluster design and COVID-related disruption may affect adherence and follow-up; supervised vs home brushing variability; biofilm and pH outcomes are surrogate markers; caries outcomes not definitively different; effect may be influenced by chitosan or formulation components rather than HAP alone |
| Kk S(1), Gangwar C(1), Sharma S(2), Kalsi R(3), Asthana G(4), Gupta V(5). | 2024 | India | Randomized, double‑blind, parallel clinical trial | Parallel-group clinical trial (42-day follow-up; examiner-blinded) | Outpatient dental setting (Public Health Dentistry clinic) | Adults aged 20-40 years with plaque-induced gingivitis (≥20 teeth; systemically healthy) | n=130 (65 per group) | Herbal dentifrice (multi-herbal formulation including neem, clove, aloe vera, Ocimum sanctum, etc.) | Toothpaste (twice daily brushing using modified Bass technique for 42 days) | Non-herbal toothpaste (fluoride-containing with triclosan and conventional ingredients) | Gingival Index (GI); Approximal Plaque Index (API); Patient Hygiene Performance (PHP index) | Significant improvements in both groups over time; herbal group showed greater reductions in GI (2.63→0.59 vs 2.65→0.71), API (51.8→24.9 vs 52.0→25.4), and improved PHP scores at 42 days (all p≤0.05); suggests modest additional benefit of herbal formulation on plaque and gingival inflammation | Short duration (42 days); behavioural confounding (oral hygiene instruction and motivation in both groups); adult gingivitis population (not caries-focused); surrogate clinical indices; heterogeneous multi-herbal formulation limits attribution of effect; limited generalisability |
| Thongmuang P(1), Thongkao K(2), Owen RW(3)(4), Sudjaroen Y(2). | 2024 | Thailand | Open‑label clinical study - skin‑irritation testing and pre/post antimicrobial assessment of UM herbal lozenges, mouth spray, and mouthwash. | Small-scale clinical evaluation with pre-post microbial assessment (pilot human study) | Clinical/real-world use (healthy volunteers; no change to routine oral hygiene practices) | Healthy adults aged 20-35 years | n=30 (microbial assessment); n=10 (skin irritation testing) | Urginea maritima (UM) herbal extract | Multiple oral care products (lozenges, mouth spray, mouthwash) | No formal comparator group (pre-post design; saline/reagent base used for irritation controls) | Oral microbial counts (modified dip-slide test): Streptococcus mutans, Candida spp., Lactobacillus spp.; product stability and skin irritation | Use of UM-containing products associated with reduction in cariogenic microorganisms (S. mutans, Candida spp., Lactobacilli); products demonstrated acceptable stability and no irritation; suggests antimicrobial potential of herbal formulations in real-world use | Very small sample size; no control/comparator group; short-term assessment; surrogate microbiological outcomes only; healthy (low-risk) population with baseline low microbial levels; no standardisation of product exposure; limited clinical relevance to caries prevention |
| Monteiro RV(1), Lins RBE(2), Lima RBW(3), Fischer K(4), De Souza GM(4). | 2024 | United States, Brazil | Scoping review (PRISMA‑ScR) | Scoping review (PRISMA-ScR; 5 databases; descriptive synthesis) | Not applicable (synthesis of clinical trials and in vitro studies in irradiated populations) | Patients with head and neck cancer undergoing radiotherapy (≥50 Gy); includes human clinical and in vitro tooth studies | n=49 included studies (36 clinical trials; 13 in vitro) | Primarily fluoride-based preventive materials (gel, toothpaste, mouthrinse, varnish); also CCP-ACP, saliva stimulants; restorative materials including resin composite, glass ionomer cement, amalgam | Various delivery formats (gel trays, toothpaste, mouth rinses, varnish; restorative placements) | Comparisons across fluoride vs non-fluoride interventions; restorative material comparisons (composite vs GIC vs amalgam) | Caries incidence/prevention; demineralisation; microbial changes; restoration survival; bond strength; material degradation; clinical outcomes over varying follow-up periods | Fluoride-based interventions (especially fluoride gel) consistently demonstrated the strongest evidence for prevention of radiation-related caries; preventive strategies (oral hygiene + fluoride) more effective than hygiene alone; glass ionomer provided protection against secondary caries but showed marginal degradation; resin composites widely used but lack strong clinical evidence in irradiated teeth | Large heterogeneity across included studies; inclusion of older studies (1970s onward); variability in methodologies, follow-up, and outcomes; limited high-quality clinical evidence for restorative materials; difficulty comparing across decades and study designs |
| Abedi M(1), Ghasemi Y(1)(2), Nemati MM(1). | 2024 | Iran | Review article - narrative (literature) review on nanotechnology in toothpaste: fundamentals, applications, trends, and safety. | Narrative review (PubMed & Web of Science search; 1995-2023) | Not applicable (review of in vitro, in vivo, and clinical studies) | Not applicable (covers general populations; includes paediatric relevance where stated) | Not applicable (review article) | Nanoparticles including hydroxyapatite (nano-HAP), calcium phosphate, calcium carbonate, sodium trimetaphosphate, titanium dioxide, zinc oxide, silver, chitosan, nanoemulsions | Primarily toothpaste formulations (also mouthwash and oral care products) | Comparisons across nanomaterials and conventional agents (e.g., fluoride vs nano-HAP; micro vs nano forms) | Remineralisation, demineralisation, hypersensitivity, antimicrobial activity, whitening, biofilm modulation, safety/toxicity | Nanoparticles enhance toothpaste efficacy via improved remineralisation, antibacterial activity, hypersensitivity reduction, and whitening; nano-HAP mimics enamel and penetrates lesions more effectively than fluoride; calcium phosphate and nano-carbonates promote remineralisation; metallic NPs (e.g., ZnO, Ag) show strong antimicrobial effects; however, evidence is heterogeneous and often preclinical | Key limitations include predominance of in vitro and laboratory-based evidence, heterogeneity in nanoparticle type, size, and formulation, limited long-term clinical data, and unresolved safety concerns regarding systemic exposure and toxicity of metallic nanoparticles |
| Nomura R(1)(2)(3), Suehiro Y(1), Tojo F(1), Matayoshi S(1), Okawa R(1)(2), Hamada M(4), Naka S(5), Matsumoto-Nakano M(5), Unesaki R(6), Koumoto K(6), Kawauchi K(6), Nishikata T(6), Akitomo T(3), Mitsuhata C(3), Yagi M(2)(7), Mizoguchi T(2)(8), Fujikawa K(2)(8), Taniguchi T(2)(7), Nakano K(1)(2). | 2024 | Japan | In vitro and small randomized crossover clinical study | In vitro experimental study with small-scale crossover clinical component | Laboratory assays (antimicrobial, growth, biofilm) + short-term human study (1-week toothpaste use) | Oral streptococci (S. mutans serotypes c/e/f/k; S. sanguinis, S. oralis, S. gordonii, S. salivarius) + healthy adults (18-30 years) | n=20 (clinical component; crossover design) | Shikonin (plant-derived extract from Lithospermum erythrorhizon) encapsulated in β-1,3-1,6 glucan dispersion | Toothpaste (5 µM shikonin; twice daily brushing for 1 week) | Shikonin-free toothpaste (crossover control) | Bacterial survival (CFU), growth inhibition, biofilm formation (confocal microscopy, OD595), salivary S. mutans counts | In vitro: strong dose-dependent inhibition of S. mutans survival (<1% viability at ≥2 µM after 6 h), growth inhibition at higher concentrations, and marked reduction in biofilm formation (≥20 µM); also inhibited non-mutans streptococci. Clinical: significant reduction in salivary S. mutans after 1 week of shikonin toothpaste use, with no change in control | Primarily in vitro evidence; clinical component very small (n=20) and short-term (1 week); surrogate microbiological outcome only; healthy young adult population; no caries or clinical endpoints; crossover design without long-term follow-up; formulation includes glucan carrier which may influence effects |
| Potocka W(1)(2), Assy Z(1)(2), Bikker FJ(1), Laine ML(2). | 2023 | Netherlands | Narrative review - monoterpenes/monoterpenoids in oral care | Narrative literature review (non-systematic) | Literature-based synthesis (no primary experimental or clinical setting) | Not applicable (compounds include monoterpenes such as thymol, eugenol, limonene, menthol, eucalyptol, carvacrol, etc.) | Not applicable | Monoterpenes and monoterpenoids (plant-derived compounds; e.g. thymol, eugenol, limonene, menthol, eucalyptol, carvacrol, hinokitiol) | Various delivery contexts (toothpastes, varnishes, essential oils, oral care products) | Not applicable (no comparator) | Biological activity (antimicrobial, anti-inflammatory, antifungal, antiviral, analgesic), mechanisms of action, potential oral health applications | Monoterpenes demonstrate broad biological activity including antimicrobial (e.g. eugenol biofilm disruption), anti-inflammatory (IL-6/IL-8 inhibition), antifungal (membrane disruption), antiviral (virion interference), and analgesic effects; several compounds already used in oral care (e.g. thymol in varnish), with potential for wider therapeutic application | Narrative (non-systematic) design; no quality appraisal; heterogeneous evidence base; largely preclinical/mechanistic data; limited direct clinical evidence; unclear dosing, safety, and long-term effects; potential publication bias |
| Navidifar T(1), Mahdizade Ari M(2)(3), Alipourkermani A(4), Afifirad R(5), Asadollahi P(6), Veisi A(7), Ghanavati R(7), Darbandi A(2). | 2022 | Iran | Systematic review (PRISMA) of randomized controlled trials | Systematic review of RCTs (PRISMA-guided; PROSPERO registered) | Mixed clinical settings (RCTs in children and adults; oral health interventions including mouthwash, dairy, lozenges, gums) | Human participants receiving probiotic interventions (n=1612 across 24 trials; ages 6-62 years) | 1612 participants (24 clinical trials) | Probiotic bacteria (commonly Lactobacillus reuteri, L. rhamnosus, Bifidobacterium spp.; also prebiotics like xylitol) | Various delivery formats (mouthwash, yoghurt/kefir, lozenges, powders, chewing gum) | Chlorhexidine (CHX), fluoride mouthwash, placebo, herbal mouthwashes | Microbial outcomes (S. mutans count, periodontal pathogens), plaque index (PI), gingival index (GI), salivary parameters, halitosis | Probiotics reduced S. mutans by ~65% on average (p<0.05) and were often equal or superior to CHX/fluoride for reducing plaque and gingival indices; also reduced periodontal pathogens (e.g. P. gingivalis, A. actinomycetemcomitans); effects varied by strain, dose, and duration | Heterogeneity in strains, doses, delivery methods, and outcome measures; variable follow-up periods; some contradictory findings (including increased S. mutans in some studies); limited standardisation; predominantly surrogate outcomes rather than clinical caries endpoints |
| Pushpalatha C(1), Suresh J(1), Gayathri VS(1), Sowmya SV(2), Augustine D(2), Alamoudi A(3), Zidane B(4), Mohammad Albar NH(5), Patil S(6). | 2022 | India / Saudi Arabia | Narrative mini‑review | Narrative (mini) review | Preclinical and translational dental material research (restorative, preventive, orthodontic, implantology, etc.) | Not applicable (review of in vitro, in vivo, and material science studies) | Not applicable | Zinc oxide nanoparticles (ZnO NPs) | Various incorporations into dental materials (composites, GIC, adhesives, dentifrices, coatings, nanogels, implants) | Not applicable | Antimicrobial activity (S. mutans, Lactobacillus, E. faecalis, etc.), remineralisation potential, dentinal tubule occlusion, mechanical properties (microleakage, flexural strength), biofilm inhibition | ZnO NPs demonstrate broad antimicrobial activity via ROS generation, membrane disruption, and ion release; when incorporated into materials they can reduce bacterial adhesion (e.g. S. mutans), decrease microleakage, enhance dentin remineralisation, and occlude dentinal tubules; also show potential in preventing secondary caries and hypersensitivity | Evidence largely limited to in vitro and laboratory-based studies; inconsistent effects on mechanical properties (e.g. reduced strength at higher concentrations due to nanoparticle aggregation); short-lived antibacterial effects in some materials; lack of robust clinical trials and long-term safety data |
| Yadav RK(1), Bharti D(1), Tikku AP(1), Verma P(1), Shakya VK(1), Pandey P(1) | 2022 | India | In vitro randomized-slab study | In vitro experimental study | Artificial caries model using extracted human molar dentine slabs (demineralisation-remineralisation cycling model) | Extracted human permanent molars (dentine slabs; artificial lesions) | n=75 (5 groups; n=15 each) | Fluoride-free toothpaste (0 ppm F), fluoride toothpaste (1000 ppm F), CPP-ACP, CPP-ACFP (CPP-ACP + fluoride), silver diamine fluoride (38% SDF) | Topical application via slurry exposure cycles with artificial saliva and acid challenge | Comparative groups (fluoride vs non-fluoride vs calcium phosphate vs SDF) | Remineralisation assessed via SEM (tubule occlusion), EDX (Ca/P ratio), QLF (fluorescence/mineral change) | SDF showed highest remineralisation (highest Ca/P ratio and tubule occlusion), followed by CPP-ACFP, CPP-ACP, fluoride toothpaste, and fluoride-free toothpaste; QLF showed greatest mineral gain for CPP-ACFP (SDF not measurable due to fluorescence interference) | In vitro model limits clinical translation; dentine slab model may not reflect in vivo oral environment; surrogate outcomes only (Ca/P ratio, fluorescence, morphology); SDF results confounded by extremely high fluoride concentration (~44,800 ppm); QLF limitations with SDF due to optical interference |
| Forcin LV(1), Oliveira TS(2), Tomaz PL(2), Matochek MH(2), Polassi MR(2), Vilhena FV(2), Svizero NR(1), D'Alpino PH(2). | 2021 | Brazil | In vitro pH‑cycling study | In vitro experimental study | pH-cycling model with brushing simulation using bovine enamel blocks | Enamel specimens (bovine; artificial caries lesions) | n=60 (divided into treatment groups; n≈8 per group) | Herbal-containing toothpastes (e.g. Galla chinensis, DOR, Herbal Bliss) with varying fluoride content (0 ppm, ~980 ppm, amine fluoride) vs NaF toothpastes (1450 ppm, 5000 ppm) | Toothpaste slurry application with brushing simulator under pH cycling | Comparison with standard fluoride toothpastes (1450 ppm, 5000 ppm NaF) | Surface hardness recovery (%SHR), cross-sectional hardness (depth remineralisation), surface roughness (Ra), physicochemical properties | All toothpastes promoted remineralisation (>100% surface hardness recovery); herbal-containing toothpastes (DOR, HBL, GCH) showed equal or greater subsurface remineralisation compared to standard fluoride toothpastes; no significant differences in %SHR between groups | Limitations include in vitro design, use of bovine enamel, pH cycling model not fully replicating oral environment, reliance on surrogate hardness outcomes, heterogeneity in toothpaste composition, and unclear contribution of individual herbal components |
| How YH(1), Yeo SK(2). | 2021 | Malaysia | Narrative literature review | Narrative review (mechanistic + translational focus) | Literature-based synthesis (oral microbiome, probiotic mechanisms, and delivery systems) | Not applicable (covers general populations; includes children and adults across cited studies) | Not applicable | Oral probiotics (e.g. Streptococcus salivarius K12, M18; Lactobacillus spp.; Bifidobacterium spp.) | Various delivery systems (lozenges, chewing tablets, capsules, oral strips, gels, mouthwash, toothpaste) | Not applicable (no formal comparator) | Microbiome modulation, pathogen inhibition, plaque reduction, gingival inflammation, delivery efficiency, adherence, viability | Oral probiotics may improve oral health via competition for adhesion sites, bacteriocin production, pH modulation (alkali production), and anti-inflammatory effects; effectiveness depends heavily on strain selection, viability, adherence, and delivery system; lozenges and tablets may enhance retention and efficacy | Narrative design; largely mechanistic and preclinical evidence; limited robust clinical data; variability in strains and formulations; effectiveness of commercial combinations often untested; issues with probiotic viability, colonisation, and standardisation |
| Courtois P(1). | 2021 | Belgium | Narrative literature review | Narrative review (mechanistic + translational) | Literature-based synthesis (salivary biochemistry, microbiology, and clinical studies) | Not applicable (oral ecosystem; includes general populations across cited studies) | Not applicable | Oral peroxidase system (sialoperoxidase, myeloperoxidase; lactoperoxidase in products) | Enzymatic system requiring substrates (H2O2 + SCN− ± I−) delivered via saliva or incorporated into oral hygiene products | Not applicable (no formal comparator) | Microbial inhibition, biofilm control, oxidative stress modulation, ecological balance, clinical indices (plaque, gingivitis in cited trials) | Oral peroxidases generate hypothiocyanite (OSCN−), an antimicrobial oxidant that inhibits bacteria, fungi, and viruses while also acting as an ecological selector favouring commensal flora; despite strong in vitro evidence, clinical efficacy of peroxidase-containing products remains poorly demonstrated due to substrate dependence, short oxidant half-life, and complex oral ecology | Primarily in vitro and mechanistic evidence; limited high-quality clinical trials; difficulty isolating peroxidase effects in vivo; dependence on substrate availability (H2O2, SCN−); variability in formulations; short-lived oxidants; challenges in measuring meaningful clinical outcomes |
| Pozos-Guillén A(1), Molina G(2), Soviero V(3)(4), Arthur RA(5), Chavarria-Bolaños D(6), Acevedo AM(7). | 2021 | Mexico, Argentina, Brazil, Costa Rica, Venezuela | Critical narrative review of international and Latin American/Caribbean evidence | Narrative review / consensus-informed critical review | Literature synthesis (clinical trials, systematic reviews, regional data) | Primary and permanent dentitions across populations (children-adults) | Not applicable | Multiple (fluoride, SDF, CPP-ACP, sealants, infiltrants, ART, restorative materials) | Range of interventions: non-invasive (fluoride, OH, diet), micro-invasive (sealants, infiltration), minimally invasive (selective caries removal, ART), restorative and pulp therapies | Not applicable (no single comparator) | Caries progression/arrest, remineralisation, restoration survival, pulp vitality, cost-effectiveness | Caries management should be biologically driven and lesion-specific, prioritising non-invasive and minimally invasive strategies (e.g. fluoride, sealants, SDF, selective caries removal) to arrest disease while preserving tooth structure; evidence supports a shift away from complete caries removal toward selective approaches and prevention-focused care, particularly in resource-limited settings | Primarily narrative synthesis; heterogeneity of included studies; limited high-quality RCTs in some areas; lack of standardisation across LACC settings; reliance on contextual adaptation and expert consensus |
| Ricomini Filho AP(1), Chávez BA(2), Giacaman RA(3), Frazão P(4), Cury JA(1). | 2021 | Argentina, Belize, Bolivia, Brazil, Chile, Colombia, Costa Rica, Cuba, Dominican Republic, Ecuador, El Salvador, Guatemala, Haiti, Honduras, Mexico, Nicaragua, Panama, Paraguay, Peru, Uruguay, Venezuela | Systematic literature review of community-level caries interventions in Latin American and Caribbean countries | Critical review with systematic search methodology (policy/epidemiological synthesis) | Literature review (PubMed, LILACS, SciELO; 37 included studies) | Population-level (children-adults across LACC countries) | Not applicable | Multiple (fluoride delivery systems, sugar restriction strategies, sealants, adjuncts like xylitol/probiotics) | Community-level interventions: water/salt/milk fluoridation, dentifrices, varnish, rinses, education, sugar replacement, sealants | Not applicable (no single comparator) | Caries prevalence/incidence, programme effectiveness, policy implementation, cost-effectiveness | Fluoride-based interventions dominate caries control strategies in LACC (26/37 studies), delivered via water, salt, milk, and dentifrices; sugar-restriction interventions are scarce and mainly limited to education, substitution (e.g. xylitol), or supplementation; effective caries control requires integrated, population-wide strategies combining fluoride exposure with upstream policies targeting sugar consumption (e.g. taxation, advertising regulation); sealants are effective but less widely implemented | Key limitations include scarcity of high-quality evidence on sugar restriction interventions, heterogeneity across countries, inconsistent surveillance of fluoride exposure (risk of fluorosis), limited coverage in rural/low-income populations, and reliance on descriptive/programmatic data rather than robust comparative trials |
| Gonçalves FMC(1), Delbem ACB(1), Gomes LF(2), Emerenciano NG(1), Dos Passos Silva M(1), Cannon ML(3), Danelon M(4)(5). | 2021 | Brazil | In vitro randomized laboratory study | In vitro experimental study using pH-cycling model | Laboratory study (bovine enamel blocks, n=72, 6 groups) | Bovine enamel model (proxy for human enamel) | 72 blocks (n=12 per group) | Fluoride (1100 ppm), CPP-ACP (MI Paste Plus®), sodium trimetaphosphate (TMP 3%) | Toothpaste slurries ± adjunct MI Paste Plus application | Placebo, fluoride alone, CPP-ACP alone, and combinations | Surface hardness loss (%SH), subsurface hardness loss (ΔKHN), lesion depth (PLM), SEM morphology, enamel Ca/P/F content | Combination 1100F-TMP-MI Paste Plus showed greatest inhibition of demineralisation (≈20-30% reduction vs fluoride alone; ≈53% reduction in subsurface lesion depth), with highest Ca and P incorporation; CPP-ACP alone showed no superiority over fluoride; TMP enhanced fluoride efficacy and facilitated ion diffusion into lesions | Key limitations include in vitro model (no saliva/biofilm), limited clinical translation, accelerated pH cycling not reflecting oral conditions, and uncertainty around long-term effectiveness and real-world application protocols |
| Juntavee A(1), Juntavee N(2), Hirunmoon P(3). | 2021 | Thailand | In vitro pH‑cycling study | In vitro experimental pH-cycling study | Laboratory study (human premolars with artificial lesions, n=60, 4 groups) | Human extracted premolars (artificial caries model) | 60 teeth (n=15 per group) | Nanohydroxyapatite (10%), tricalcium phosphate (TCP), fluoride (≈1000-1100 ppm) | Toothpaste slurry application twice daily during 10-day pH-cycling model | No treatment control; comparisons between nano-HA, TCP, and fluoride | Surface microhardness (VHN), % hardness recovery (%HR), remineralisation potential (%RP), lesion depth (PLM), SEM morphology, XRD crystallinity | All three toothpastes significantly improved remineralisation vs control; no significant difference in %HR or %RP between nano-HA, TCP, and fluoride; however, nano-HA showed greater lesion depth reduction (PLM) and more homogeneous surface remineralisation (SEM), suggesting enhanced structural repair despite similar hardness outcomes | Key limitations include in vitro design (no saliva/biofilm), short experimental duration, artificial lesions, and limited translation to clinical conditions |
| Ranjana BS(1), Chowdhary N(1), Kiran NK(1), Chaitan SM(1), Reddy VR(1), Prabahar T(1). | 2021 | India | In vitro pH‑cycling study | In vitro experimental study (pH-cycling model) | Laboratory study using extracted primary teeth (artificial lesions) | Primary anterior teeth (demineralised enamel sections) | n=60 sections (from 30 teeth; ~27 per group) | Non-fluoride dentifrice (TCP-based, Mee Mee®) vs herbal dentifrice (Aloe vera + xylitol, Mamaearth™) | Dentifrice slurry exposure within 7-day pH cycling model | Direct comparison: non-fluoride vs herbal dentifrice | Lesion depth (µm) via polarized light microscopy (PLM) and image analysis | Non-fluoride dentifrice showed significantly greater remineralisation (mean lesion depth 7.87 µm) compared to herbal dentifrice (28.31 µm); lower lesion depth indicates greater remineralisation; difference statistically significant (p=0.000) | Key limitations include in vitro design, short duration (7 days), absence of saliva and biofilm, small sample size, and limited generalisability to clinical settings |
| Degli Esposti L(1), Ionescu AC(2), Brambilla E(2), Tampieri A(1), Iafisco M(1). | 2020 | Italy | Materials characterization (PXRD, FTIR‑ATR, ICP‑OES, TGA) plus short‑term in vitro human tooth tests | In vitro experimental biomaterials study (SEM, EDX, PXRD, FTIR analyses) | Laboratory study using extracted human teeth (enamel and dentine specimens) | Human molar enamel and dentine specimens (acid-demineralised) | ~18 treated specimens + controls (enamel and dentine) | Biomimetic hydroxyapatite (carbonate-, Mg-, Sr-, F-doped) + silica + chitosan + fluoride (~1450 ppm) | Toothpaste brushing protocol (3× daily for 3 days) vs water control | Control: demineralised specimens treated with water only | Surface morphology (SEM), elemental composition (EDX), crystallinity (PXRD), chemical structure (FTIR), dentinal tubule occlusion | Toothpaste induced remineralisation via deposition of a new apatite-like crystalline phase aligned with native enamel (epitaxial growth); interprismatic spaces filled; dentinal tubules completely occluded; incorporation of F and Sr into surface layer; strong qualitative remineralisation effect demonstrated | Key limitations include in vitro design, short treatment duration (3 days), qualitative/semiquantitative outcomes, lack of biofilm/saliva simulation, and absence of clinical validation |
| Vilela MM(1), Salvador SL(2), Teixeira IGL(3), Del Arco MCG(2), De Rossi A(3). | 2020 | Brazil | Randomized clinical trial in children | Randomised clinical trial (parallel groups, microbiological outcomes) | Paediatric dental clinic (clinical + laboratory microbiology processing) | Children aged 5-12 years at high caries risk (≥3 active lesions; ≥10^6 CFU/mL baseline) | n=47 (EGCG n=15; green tea n=15; CHX n=15; water n=7) | Epigallocatechin-3-gallate (EGCG, 4000 μg/mL); green tea (Camellia sinensis infusion) | Mouthrinse (3 mL for 1 minute; single exposure; saliva sampled at baseline and 10 min post-rinse) | Positive control: chlorhexidine 0.12%; Negative control: distilled water | CFU/mL of mutans streptococci and lactobacilli (pre- vs post-rinse); % microbial reduction | All groups showed significant reductions post-rinse; EGCG achieved greater reduction than green tea and water but less than CHX; mutans streptococci reduction: EGCG 79.9%, green tea 68.3%, water 50.6%, CHX 95.5%; lactobacilli reduction: EGCG 72.1%, green tea 59.2%, water 42.0%, CHX 86.0% | Key limitations include single short-term exposure (10 min outcome), small sample size, reliance on CFU counts and morphological identification, lack of blinding detail, no long-term clinical outcomes (caries incidence), and no biofilm-level assessment |
| Polyakova MA(1), Arakelyan MG(1), Babina KS(1), Margaryan EG(1), Sokhova IA(1), Doroshina VY(1), Novozhilova NE(1). | 2020 | Russia | Randomized double‑blind clinical trial | Randomised, double-blind, controlled clinical trial (4-week follow-up) | University dental clinic (young adult population) | Adults aged 20-25 years with enamel hypersensitivity | n=60 (brushite n=20; HAP n=20; negative control n=20) | Brushite-forming toothpaste (Ca/P system forming dicalcium phosphate dihydrate crystals) | Toothbrushing twice daily for 4 weeks | Positive control: hydroxyapatite toothpaste (6%); Negative control: non-remineralising toothpaste | Primary: Schiff sensitivity index; Secondary: enamel acid resistance (staining score), remineralisation rate (days to loss of staining), OHI-S | Both brushite and HAP groups showed significantly increased enamel acid resistance and faster remineralisation vs control; HAP showed earlier effect (2 weeks), brushite significant at 4 weeks; hypersensitivity reduced significantly in both groups vs control; no significant difference between brushite and HAP overall | Key limitations include narrow age range (20-25 years), short follow-up (4 weeks), surrogate outcomes (staining-based remineralisation), and limited generalisability to broader populations |
| Khoramian Tusi S(1), Jafari A(2)(3), Marashi SMA(4), Faramarzi Niknam S(1), Farid M(5), Ansari M(6). | 2020 | Iran | Randomized, double‑blind crossover clinical trial | Randomised, double-blind, crossover clinical trial (AB/BA design) | Dental school setting (controlled clinical + microbiological analysis) | Healthy dental students aged ~21-26 years (no active caries) | n=22 (crossover; each participant receives both intervention and placebo) | Teucrium polium herbal extract mouthwash (0.2%) | Mouthrinse (15 mL for 30 seconds, twice daily for 2 weeks; crossover with 3-week washout) | Placebo mouthwash (no active extract) | Primary: S. mutans CFU/mL (saliva samples pre- and post-intervention phases) | Teucrium polium mouthwash significantly reduced S. mutans counts in both phases (p<0.001), while placebo showed no significant reduction; antimicrobial effect persisted beyond washout period; crossover analysis confirmed treatment effect without carryover bias | Key limitations include small sample size, surrogate microbiological outcome (CFU), short intervention duration, absence of clinical caries outcomes, and lack of comparison with gold-standard agents such as chlorhexidine |
| Gouvêa DB(1), Santos NMD(1), Pessan JP(2), Jardim JJ(3), Rodrigues JA(1). | 2020 | Brazil | In vitro study using pH‑cycling and transverse microradiography | In vitro experimental pH-cycling study with TMR analysis | Laboratory study using human permanent and deciduous enamel blocks | Human enamel (permanent and primary teeth; artificial subsurface lesions) | n≈20-30 blocks across phases (grouped by enamel type and treatment) | Fluoride dentifrice (1450 ppm NaF) vs fluoride-free dentifrice | Toothpaste slurry treatment during 10-day pH-cycling model (solution “B” with low fluoride content) | Direct comparison: fluoridated vs non-fluoridated dentifrice | Lesion depth (µm), integrated mineral loss (ΔZ), mineral distribution (R-values) via transverse microradiography (TMR) | Solution “B” successfully produced subsurface lesions (~88-89 µm initial depth); fluoride dentifrice significantly reduced lesion progression and mineral loss compared to fluoride-free dentifrice (p<0.05); fluoride-free groups showed substantially greater lesion depth increase (~73-75 µm vs ~22 µm with fluoride) and higher ΔZ (greater mineral loss) | Key limitations include in vitro design, artificial lesion model, accelerated pH cycling, lack of biofilm/saliva, and reliance on surrogate mineral outcomes rather than clinical endpoints |
| Wierichs RJ(1), Musiol J(2), Erdwey D(3), Esteves-Oliveira M(4), Apel C(5), Meyer-Lueckel H(6). | 2020 | Germany | Double-blind, randomized cross-over in situ trial | Double-blind, randomised, crossover in situ study (4×4-week arms) | In situ intraoral appliance model (human participants wearing mandibular appliances with enamel and dentine specimens) | Healthy adults (n=20) wearing appliances containing enamel and dentine specimens (sound + artificial lesions) | n=20 participants; ~405 analysable specimens (enamel + dentine) | Nano-hydroxyapatite toothpaste (0 ppm F) vs fluoride dentifrices (0 ppm, 1100 ppm, 5000 ppm NaF) | Toothbrushing with slurry applied to specimens twice daily over 4-week periods | Negative control: fluoride-free toothpaste; Positive controls: 1100 ppm and 5000 ppm fluoride dentifrices | Primary: change in integrated mineral loss (ΔΔZ); Secondary: lesion depth (ΔLD), TMR mineral density profiles | Fluoride dentifrices (1100 and 5000 ppm) showed significant remineralisation across dentine and enamel; clear fluoride dose-response observed (strongest in highly demineralised dentine); nano-hydroxyapatite toothpaste showed no significant difference from fluoride-free control and did not inhibit demineralisation | Key limitations include in situ (not fully clinical) model, use of bovine specimens, indirect outcomes (TMR), and influence of model conditions (e.g., sucrose exposure, appliance-based biofilm) |
| Hobbs M(1), Marek L(2), Clarke R(3), McCarthy J(3), Tomintz M(2), Wade A(4), Campbell M(2), Kingham S(2). | 2020 | New Zealand | Cross‑sectional analysis of nationally representative 2017/18 New Zealand Health Survey data | Cross-sectional observational study (national survey analysis) | New Zealand Health Survey 2017/18 (population-based, multistage sampling) | Children (n=4,723) and adults (n=13,869) nationally representative sample | n=18,592 total participants | Not an intervention study (exposure: type of toothpaste used) | N/A (self-reported toothpaste use) | Comparisons across age, ethnicity, socioeconomic status, rural/urban classification, and geography (DHBs) | Primary: prevalence of non-fluoride toothpaste use; Secondary: sociodemographic associations and spatial variation | Non-fluoride toothpaste use reported in 6.4% of children and 6.8% of adults; higher prevalence in Asian populations and in moderately to least deprived areas; lower prevalence in most deprived groups; spatial variation observed across regions; majority still used fluoride toothpaste (≈80-85%) | Key limitations include self-reported data, lack of linkage to clinical outcomes, cross-sectional design (no causality), and potential misclassification of toothpaste type |
| Carrouel F(1), Viennot S(1), Ottolenghi L(2), Gaillard C(3), Bourgeois D(1). | 2020 | France; Italy | Narrative literature review | Narrative review (structured database search) | Literature from PubMed, Web of Science, and Scopus (2000-2019) | N/A (review of multiple in vitro, in vivo, and clinical studies) | N/A (review paper) | Multiple nanoparticle classes (e.g. silver, gold, zinc oxide, titanium dioxide, hydroxyapatite, CPP-ACP, bioactive glass, chitosan, chlorhexidine NPs) | Various delivery systems (toothpaste, mouthwash, toothbrushes, varnishes) | Comparators vary by included studies (e.g. fluoride, chlorhexidine, conventional formulations) | Anti-microbial activity, anti-inflammatory effects, remineralisation capacity, biofilm disruption, dentine hypersensitivity reduction | Nanoparticles demonstrate multi-functional roles: (1) anti-microbial via ROS generation, membrane disruption, and ion release; (2) anti-inflammatory via cytokine modulation; (3) remineralisation via biomimetic deposition (e.g. hydroxyapatite, CPP-ACP, bioactive glass); silver and zinc oxide show strong anti-bacterial effects, hydroxyapatite shows biomimetic enamel repair; nanoparticle effects are size-, morphology-, and concentration-dependent | Key limitations include heterogeneity of included studies, predominance of in vitro data, unclear long-term safety/toxicity, lack of standardisation in nanoparticle synthesis and application, and limited clinical outcome data |
| Cocco F, Salerno C, Wierichs RJ, Wolf TG, Arghittu A, Cagetti MG, Campus G | 2025 | Italy; Switzerland; Germany; India; Sweden | Triple‑blind, randomized, parallel‑group clinical trial | Triple-blind randomised clinical trial (24-month follow-up) | University dental clinic + school-based setting (Sassari, Italy; multicentre academic collaboration) | Children aged 4-7 years with active caries lesions in primary dentition | n=610 enrolled; n=518 completed (per-protocol) | Hydroxyapatite-fluoride (HAF) toothpastes (1000 ppmF and 1450 ppmF; ion-doped HA in chitosan matrix) | Toothbrushing 3× daily for 2 minutes over 24 months | Comparator: sodium monofluorophosphate (NaMFP) toothpastes (1000 ppmF and 1450 ppmF) | Caries activity status (active, inactive, partially active), enamel and dentine lesion progression/arrest (visual-tactile ICDAS-based assessment) | HAF group showed significantly greater reduction in enamel lesion activity vs NaMFP (P<0.01); higher proportion of active lesions became inactive (≈74% in HAF); dentinal lesion outcomes similar but with greater inactivation in HAF group (P=0.03); overall greater caries arrest and remineralisation in HAF group | Key limitations include COVID-related follow-up disruption, per-protocol analysis (attrition bias), visual-tactile assessment only (no radiographs), complex intervention (HA + fluoride), and difficulty isolating mechanism of effect |
| Luo W, Lee GHM, Wong H, Wong M | 2022 | Hong Kong (China) | Double-blind parallel-arm randomised controlled trial | Double-blind parallel-arm randomised controlled trial (2-year follow-up; embedded within PhD thesis with systematic review component) | Community-based (parent-infant dyads; oral health programme with monitoring and reinforcement) | Young children (~14 months at baseline; high caries risk) | n=472 randomised (xylitol n=240; fluoride n=232); baseline cohort n=579 parent-infant dyads | 25% xylitol toothpaste | Toothbrushing twice daily for 2 years | Comparator: 1000 ppm fluoride toothpaste | Caries incidence at 2 years; visual plaque index; S. mutans levels (microbiological outcomes) | No significant difference in caries incidence between groups (xylitol 9.2% vs fluoride 9.9%; RR 0.92, 95% CI 0.50-1.69; P=0.876); xylitol group showed lower plaque index (P=0.034); xylitol may modulate S. mutans levels but without clinical superiority | Key limitations include young age cohort (low baseline caries incidence), potential underpowering for clinical differences, behavioural co-intervention (oral hygiene reinforcement), and difficulty isolating independent effect of xylitol |
| Priyal G, Jose M, Nayak S, Pai V, Prabhu S | 2021 | India | Randomized, parallel‑group, open‑label in vivo clinical trial | In vivo experimental study (parallel group design, 6-week follow-up) | Dental college setting (controlled oral hygiene conditions) | Healthy young adults aged 18-20 years | n=120 (6 groups of 20 participants; 4 herbal, 2 non-herbal toothpastes) | Multiple formulations: herbal (neem, miswak, babool, mango leaf extracts) and non-herbal (triclosan; sodium monofluorophosphate ± triclosan) | Toothbrushing twice daily for 6 weeks with assigned toothpaste | Comparator: between-group comparison of different toothpaste formulations (no true placebo control) | Primary: microbial load (CFU/mL) from subgingival plaque; Secondary: presence of specific organisms (S. mutans, S. mitis, S. salivarius, anaerobes) | All toothpaste formulations (herbal and non-herbal) demonstrated reductions in microbial load over time; however, no statistically significant differences between groups overall (p>0.005); some individual formulations showed organism-specific reductions (e.g. babool vs S. mutans), but findings were inconsistent across groups | Key limitations include short duration (6 weeks), surrogate microbiological outcomes (CFU), lack of true control group, small group sizes, and inability to isolate active ingredient effects due to multiple formulations |
| Parkinson CR, Butler A, Ling MR | 2023 | India; China; USA | Pooled analysis of six randomized, examiner‑blind, parallel‑group controlled clinical trials | Pooled analysis of six randomized controlled trials (6-24 weeks duration; patient-level data) | Multicentre (clinical trials conducted across India, China, and USA; dental clinical settings with prophylaxis at baseline) | Adults ≥18 years with mild-moderate gingivitis | n=1601 randomised (640 sodium bicarbonate; 641 control; 320 additional arm); n=1474 completed | 67% sodium bicarbonate toothpaste (with fluoride 932-1450 ppm) | Twice-daily toothbrushing for 6-24 weeks following professional prophylaxis | Comparator: non-sodium bicarbonate (regular fluoridated toothpaste) | Primary: number of bleeding sites; Secondary: bleeding index (BI), modified gingival index (MGI), plaque index (TPI) | Sodium bicarbonate toothpaste showed statistically significant improvements vs control across all outcomes (p<0.0001); at 24 weeks, bleeding sites reduced from ~56 to ~16 (≈48% reduction vs control); improvements observed across all tooth regions and sites, with greatest effects in facial and papillary regions; consistent benefit across countries | Key limitations include industry funding (Haleon), pooled analysis restricted to company-conducted trials, heterogeneity in study duration/design, and applicability limited to populations receiving professional prophylaxis and compliant oral hygiene |
| Passos VF, Sousa RBRA, de Melo MAS, Gomes EAB, Santiago SL, Lima JPM | 2021 | Brazil | Randomized, in vitro erosion-abrasion study | In vitro experimental study (erosion-abrasion cycling model) | Laboratory study using extracted primary teeth enamel slabs | Primary (deciduous) enamel specimens (from extracted caries-free molars) | n=30 teeth (~90 enamel slabs; n=8 per group analysed) | Fluoride toothpaste (1100 ppm NaF) vs fluoride-free toothpaste | Brushing abrasion following erosive challenge (orange juice, pH 3.38; 3× daily for 5 days) | Control: distilled water (no toothpaste) | Primary: enamel surface loss (µm) via stylus profilometry | Fluoride toothpaste showed significantly less enamel loss (≈1.88-2.14 µm) compared with fluoride-free toothpaste (≈3.32-3.62 µm; p=0.04); no significant difference between fluoride toothpaste and water; fluoride provides partial protection against erosion-abrasion | Key limitations include in vitro design, short experimental duration (5 days), artificial erosion model (orange juice), small sample size, and limited clinical translation to real-world paediatric settings |
| Creeth JE, Burnett GR, Souverain A, Gomez-Pereira P, Zero DT, Lippert F, Hara AT | 2020 | United Kingdom; Switzerland; USA | Randomized, investigator‑ and specimen‑analyst‑blind, three‑period, three‑treatment in situ crossover trial | Randomised, investigator-blind, crossover in situ clinical trial (3-period design) | Single-centre in situ model (Indiana University Oral Health Research Institute; palatal appliance with enamel specimens) | Healthy adults (18-65 years) wearing palatal appliances with bovine enamel specimens | n=62 completed (randomised crossover design) | Experimental sodium fluoride toothpaste (1150 ppm F; with lactate and PVM/MA copolymer) | Single brushing episode with intra-oral exposure (2 h and 4 h), followed by in vitro acid challenge | Comparators: stannous fluoride (SnF2-Zn) toothpaste (1100 ppm F) and fluoride-free placebo | Primary: surface microhardness recovery (SMHR); Secondary: relative erosion resistance (RER), enamel fluoride uptake (EFU), acid resistance ratio (ARR) | Test (NaF) toothpaste showed significantly greater enamel rehardening, fluoride uptake, and resistance to demineralisation vs both SnF2 and placebo at 2 h and 4 h (p<0.0001); superior SMHR, RER, and EFU; ARR superior at 2 h vs both comparators and at 4 h vs placebo only | Key limitations include in situ model (not fully clinical), short-term exposure, use of bovine enamel specimens, and industry funding (GSK), which may introduce bias |
| Panchbhai, Aarati S.1; Khatib, Mahalaqua Nazli2; Borle, Rajiv M.1; Deolia, Shravani S.1; Babar, Vijay M.2; Vasistha, Anjali H.1; Parida, Ritika P. | 2024 | India | Systematic review and meta‑analysis | Systematic review and meta-analysis of randomized controlled trials (PROSPERO registered; PRISMA-guided) | Multiple settings across included RCTs (community, school-based, clinical) | Healthy preschool children aged 1-6 years (with or without caries) | 9 RCTs included (sample sizes vary across studies) | Probiotics (various strains including Lactobacillus, Bifidobacterium, Streptococcus; delivered via milk, yogurt, tablets, lozenges, etc.) | Varied across studies (weeks to months; differing delivery formats and dosing) | Comparators: placebo, no treatment, or alternative preventive interventions | Primary: caries incidence (dmft/DMFT, ICDAS); Secondary: S. mutans levels, plaque index, pH, adverse effects | Meta-analysis suggests probiotics reduce cavitated lesions (mean difference −0.44 [−0.89, 0.01]) and may reduce caries prevalence; effects on S. mutans reduction inconsistent and often non-significant; overall findings described as “encouraging” but evidence remains limited and heterogeneous | Key limitations include heterogeneity of probiotic strains, delivery methods, and study duration; small sample sizes; variable risk of bias across included studies; and lack of high-quality long-term clinical evidence |
| Schlagenhauf, U., Kunzelmann, K.H., Hannig, C., May, T.W., Hösl, H., Gratza, M., Viergutz, G., Nazet, M., Schamberger, S. and Proff, P | 2020 | Germany | Multicenter, double‑blind, randomized, parallel‑group non‑inferiority trial | Randomised, double-blind, multicentre non-inferiority clinical trial (6 months) | Orthodontic clinical settings (multicentre: Wuerzburg, Regensburg, Munich, Dresden, Frankfurt) | Adolescents and young adults (11-25 years) undergoing fixed orthodontic treatment with high caries risk (elevated S. mutans) | n=150 recruited; n=147 ITT; n=133 per protocol | 10% microcrystalline hydroxyapatite toothpaste (fluoride-free) | Twice-daily toothbrushing for 168 days with standardised electric toothbrush | Comparator: fluoridated toothpaste (amine fluoride 350 ppm + stannous fluoride 1050 ppm ≈1400 ppm total) | Primary: new enamel caries lesions (ICDAS ≥1); Secondary: ICDAS ≥2 lesions, plaque index (PI), gingival index (GI) | HAP toothpaste was non-inferior to fluoride control; no significant differences in caries progression (ICDAS ≥1: 54.7% vs 60.9% PP; ICDAS ≥2: 23.4% vs 34.8% PP); plaque and gingival indices increased over time in both groups with no between-group differences | Key limitations include non-inferiority design (not superiority), absence of negative control, high-risk orthodontic population limiting generalisability, short follow-up (6 months), and industry funding |
